# Supplementary material for: A biodegradable hybrid inorganic nanoscaffold for advanced stem cell therapy
Source: Nat Commun. 2018 Aug 8;9:3147. doi: 10.1038/s41467-018-05599-2 (PMC6082841; doi:10.1038/s41467-018-05599-2)
Supplement: Supplementary file 1 — Supplementary Information [file 41467_2018_5599_MOESM1_ESM.pdf]

SUPPORTING INFORMATION:

(Submitted to *Nature Communications*)

A Biodegradable Hybrid Inorganic Nanoscaffold for Advanced Stem Cell Therapy

Letao Yang[a], Sy-Tsong Dean Chueng[a], Ying Li[b], Misaal Patel[b], Christopher Rathnam[a], Gangotri Dey[a], Lu Wang[a], Li Cai[b] and Ki- Bum Lee[a, b*]

[a] *Department of Chemistry and Chemical Biology, Rutgers, The State University of New Jersey*

123 Bevier Road, Piscataway, NJ 08854, USA

[b] *Department of Biomedical Engineering, Rutgers, The State University of New Jersey*

599 Taylor Road, Piscataway, NJ 08854, USA

***Correspondence:**

Prof. Ki-Bum Lee

Department of Chemistry and Chemical Biology, Rutgers, The State University of New Jersey
123 Bevier Road, Piscataway, NJ 08854, USA
Tel. +1-732-445-2081; Fax: +1-732-445-5312
E-mail: kblee@rutgers.edu
http://kblee.rutgers.edu/

TABLE OF CONTENTS

A. Methods and Experimental details

B. Supplementary (SI) Figures, Tables and Video

SI 1. Characterizations of 2D MnO_2 nanosheets

SI 2. Modeling nanosheet-small molecule interactions using DFT calculation

SI 3. Characterizations of MnO_2 hybrid nanoscaffold

SI 4. Vacuum filtration approach for fabricating MnO_2 and GO hybrid nanoscaffold

SI 5. Biocompatibility of MnO_2 hybrid nanoscaffold

SI 6. Enhanced neuronal differentiation of iPSC-NSCs on MnO_2 hybrid nanoscaffold

SI 7. Representative immunostaining images of iPSC-NSCs differentiated on MnO_2 laminin nanoscaffolds using mature neuronal markers (MAP2 and Synapsin 1)

SI 8. Laminin concentration-dependent adhesion of iPSC-NSCs on different scaffolds

SI 9. Tunable biodegradability of MnO_2 nanoscaffold

SI 10. Tuning biodegradability of MnO_2 nanoscaffolds by modulating scaffold structure and varying cell densities.

SI 11. iPSC-NSCs adhesion on MnO_2 laminin hybrid nanoscaffold

SI 12. Drug (RhB) daily releasing profiles from MnO_2 laminin hybrid nanoscaffold and control polymer (PCL) scaffold

SI 13. Drug release profile of DAPT loaded on MnO_2 nanoscaffold

SI 14. Loading of neurogenic drug on MnO_2 laminin hybrid nanoscaffolds

SI 15. Short-term neuronal differentiation of iPSC-NSCs on DAPT-loaded MnO_2 laminin hybrid nanoscaffold.

SI 16. Long-term neuronal differentiation of iPSC-NSCs on DAPT-loaded MnO_2 laminin hybrid nanoscaffold.

SI 17. *In vivo* biocompatibility and biodegradability of MnO_2 laminin hybrid nanoscaffolds

SI 18. Representative mice images 3-week post-transplantation

SI 19. Cell proliferation in the MnO_2 laminin hybrid nanoscaffolds

SI 20. Supplementing animal groups for studying the effects of MnO_2 nanoscaffold ~~and~~ on enhanced transplantation of iPSC-NSCs

SI 21. Immunohistological images demonstrating effects on endogenous NSC neurogenesis

SI 22. Immunohistological staining images demonstrating the effects on astroglial cell intensities

SI 23. Enhanced cell survival of transplanted GFP-iPSC-NSCs on MnO_2 nanoscaffolds

SI 24. Enhanced transplantation and neuronal differentiation of iPSC-NSCs by 3D BHI nanoscaffold 1-month post injury.

SI 25. Supporting images for nanoscaffold and control treated Notch1CR2 GFP mice

SI Table 1. Summary of binding energies between small molecules and MnO_2

SI Table 2. Summary of the primers used for quantitative PCR

SUPPLEMENTARY METHODS

Degradation of micro-contact patterned MnO_2 nanoscaffolds: We first generated Photoresist (PR) micropatterns on Silicon wafer based on photolithographic technique. Then silicon coated with photoresist micropattern was deposited with a layer of (heptadecafluoro-1,1,2,2-tetrahydrocecyl)trichlorosilane for 2 h in a desiccator. PDMS (Sylgard 184 silicone elastomer base and curing reagent) was then poured into pre-coated photoresist patterns and kept in a 60 °C oven for curing. The PDMS mold with micro-patterns was then detached from photoresist and treated with oxygen plasma ($1.5*10^{-1}$ Torr, 25 seconds). MnO_2 nanosheet solution at 2.0 mg per ml was drop-casted on PDMS stamp and spin coated at 500 rpm for 10 seconds, 1500 rpm for 20 seconds and 3000 rpm for 30 seconds. At the same time, a glass substrate (please note that gold and silicon also work under the same parameters) was treated with oxygen plasma (Femto Science, Cute series) for 1 minute. Then the PDMS stamp coated with the MnO_2 solution was tilted and pressed on the glass substrate. After 1 minute, a pressure of 75 g per cm^2 was mounted on PDMS stamps for 20 minutes. The MnO_2 nanosheet patterned glass was finally washed with ethanol and water and imaged under an optical microscope. To monitor the degradation of MnO_2 nanosheet micropatterns on the glass, the substrate was incubated with ascorbic acid (50 g per ml) solution and images were taken before and after solution treatment. Field Emission Scanning Electron Microscopy (FESEM, Zeiss Sigma) was used for micrograph acquisition, and Electron Dispersive X-ray (EDX) was also used to surface changes of the substrate before and after ascorbic acid treatment under identical parameters.

Measurement of MnO_2 nanosheet degradation in solution: A series of PBS solutions containing different ascorbic acid concentrations (Sigma-Aldrich, 0 µg per ml, 10 µg per ml, 50 µg per ml, 100 µg per ml, 200 µg per ml, 500 µg per ml) were first prepared. Into 3 ml solutions of each solution, 10 µL of 3 mg per ml MnO_2 nanosheet solution was added, and the absorbance peak of MnO_2 nanosheet solution at 385 nm was used to quantify the amount of MnO_2 nanosheet remaining in the solution. The measurement was taken at one-minute interval for 10 minutes. The percentage of MnO_2 nanosheet remaining was calculated by normalizing to

the MnO_2 nanosheet absorption intensities at 385 nm at PBS solutions (MnO_2 nanosheet concentration of 0 μg per ml).

Covalent conjugation on the MnO_2 nanoscaffold: To study the ability of nanoscaffold for covalent conjugation, an ethanolic solution of Amino Propyl triethoxysilane (APTES, Sigma-Aldrich) was used for conjugation. Nanoscaffold was incubated in 1.0 mg per ml APTES solution for 1 hour and then washed with ethanol and water for 3 times each. Then the APTES functionalized substrate was incubated in 1.0 mg per ml fluorescamine (Sigma-Aldrich) solution (ethanol solution) for 4 hours. Fluorescamine selectively reacts with an amine group on the APTES functionalized nanoscaffold and forms a fluorescent compound. After washing with ethanol and water for a few times, the substrate was imaged under a fluorescent microscope. The blue fluorescence from the substrate indicates the chemical functionality of nanoscaffold toward APTES.

Human Neural Progenitor Cell (hNPC) Culture: The human neural progenitor cell (hNPC) line was purchased from Millipore (Catalog No.: SCC008) and cultured according to the manufacturer's protocol. Cell line was authenticated by ReNeuron Group plc and validated by high expression of Sox2 and Nestin. Tissue culture vessel were treated with 20μg per cm^2 of Poly-L-Lysine (Sigma-Aldrich) and 7 μg per cm^2 laminin (Life Technologies) for 6hrs at room temperature and 4hrs at 37°C respectively. All cells were maintained at 37°C in a humidified incubator with 5% CO_2. All experiments were conducted on passage number 3 and 5 cells. The hNSCs were seeded at 33K cells per cm^2 in hNPC specific media (from Millipore) supplemented with basic fibroblast growth factor (bFGF, 20 ng per mL) and epidermal growth factor (EGF, 20 ng per mL) with media exchanges occurring every other day. Neural differentiation of hNPC is done with the withdrawal of bFGF and EGF. Through qPCR, TuJ1 gene is checked for successful neuronal differentiation.

Immunocytochemistry: To confirm neuronal differentiation, we conducted immunocytochemistry study. All fluorescence images were obtained using a Nikon Eclipse Ti-E inverted fluorescence microscope. Following the generation of mature neurons, media was removed, and the cells were fixed for 15 minutes in formalin (Sigma) followed by two washes with D-PBS. The nucleus was stained with DAPI (Life Technologies, Catalog Number:

D1306, 1:100 dilution) for 30 minutes and then washed with PBS three times. Cells were then permeabilized with 0.1% Triton X-100 in PBS for 10 minutes, and non-specific binding was blocked with 5% normal goat serum (NGS, Life Technologies) in D-PBS for 1 hr at room temperature. Neuronal marker TuJ1 was stained using the mouse monoclonal antibody against TuJ1 (Biolegend, catalog number: 801202, 1:500 dilution). Mature neuronal markers MAP2 (mouse monoclonal, Cell Signaling, catalog number: 8707S, dilution factor: 1:500), and Synapsin 1 (rabbit monoclonal, Sigma Aldrich Millipore, catalog number: AB1543P, 1:500 dilution) were also used for the immunostaining analysis (SI Figure 7). Following the manufacturer's protocol, the fixed samples were incubated overnight at 4°C in a solution of these antibodies in PBS containing 10% NGS. After washing three times with PBS, the samples were incubated for 1 hr at room temperature in a solution of Alexa 594 anti-mouse secondary antibody (Biolegend, catalog number: 8890, 1:300 dilution) and Alexa 488 anti-rabit secondary antibody (Biolengend, Catalog Number:406416, 1:300 dilution), in PBS containing 10% NGS, and washed with D-PBS three times thereafter. For tissue and cell culture analysis, all GFP+, TuJ1+, MAP2+ cells were identified by automatic function in the Nikon NIS element software and then amounts of cells were recorded.

Focal adhesion kinase studies: To study the effect of laminin density on the adhesion of iPSC-NSC, MnO_2 nanoscaffold, GO nanoscaffold and glass were coated with laminin under identical conditions except for the concentrations (5 μg per ml, 10 μg per ml, 20 μg per ml) during laminin solution coating. After 3 days' differentiation process, the morphology and attachment of cells were imaged under an optical microscope. To further quantify focal adhesion kinase that is directly involved in the cell adherence, cells cultured on different substrates and different laminin concentrations were trizoled. FAK mRNAs and GAP43 mRNAs were then quantified and normalized to GAPDH mRNAs in the qRT-PCR analysis.

Gene Expression Analysis: Total RNA was extracted using TRIzol Reagent (Life Technologies) and transcribed to cDNA for quantitative PCR (qPCR) analysis. Specifically, cDNA was generated from 1 μg of total RNA using the Superscript III First-Strand Synthesis System (Life Technologies). The qPCR reactions

were performed using Power SYBR Green PCR Master Mix (Applied Biosystems) on a StepOnePlus Real-Time PCR System (Applied Biosystems) with the primers specific to each of the target mRNAs. The resulting Ct values were normalized to GAPDH. Standard cycling conditions were used for all reactions with a melting temperature of 60 C. All primers were obtained from the PrimerBank database, purchased from IDT Technologies and listed in SI Table 2.

Immunohistochemistry: Frozen spinal cord tissue was sectioned transversely or sagittally (10-12 µm in thickness) using a cryostat (ThermoScientific) and air dried. Sections were blocked and permeablized for 1 hr in blocking buffer containing 10% donkey serum, 0.1% TritonX, and 0.1% Tween® 20 at room temperature. Afterwards, they were incubated with primary antibodies overnight at 4℃. Following three 10-min washes in PBS, sections were incubated in the blocking buffer containing corresponding fluorophore-conjugated secondary antibodies for 1 hour at room temperature. Slides were then washed for three times with PBS (10-min each), stained with DAPI and then incubated with 4 mM $CuSO_4$ in 50 mM ammonium acetate buffer for 10 min to reduce autofluorescence. Sides were then mounted with mounting media (Vector Laboratories) right away, and images were taken within a week. The following primary antibodies were used: PH3 (1:100, rabbit polyclonal, sc-8656-R) from Santa Cruz Biotechnology, TuJ1, cleaved Caspase 3 (Cell Signalling, Catalog number: 9661T, 1:500 dilution), GFAP (1:1000 dilution, mouse monoclonal, G3893) from Sigma Aldrich and F4/80 (1:500 dilution, ab16911) from Abcam. Alexa 488 conjugated anti-rabbit and Alexa 594 conjugated anti-mouse antibody from Biolegend were used at 1:500 dilutions. Images were captured and analyzed using a Zeiss Axio Imager M1 fluorescence microscope and visualized with AxioVision 4.8 and Nikon Eclipse Ti-E microscope.

SUPPLEMENTARY FIGURES

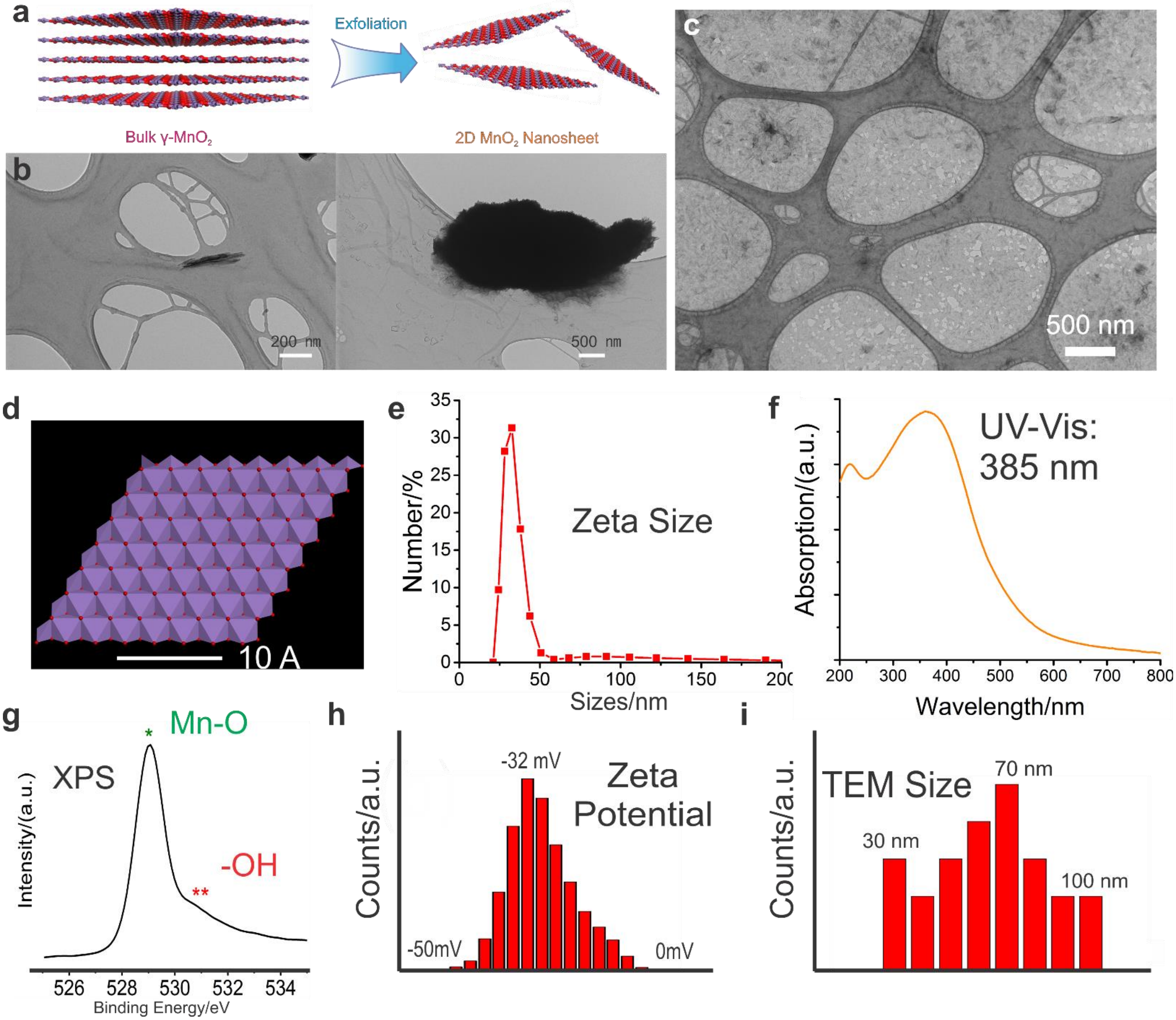

Supplementary Figure 1. Characterizations of 2D MnO_2 nanosheets. a-b, Schematic diagram explaining the synthesis of 2D anisotropic MnO_2 nanosheet from birnessite-type of MnO_2 precursors, and representative TEM images of the precursor with a layered structure. Birnessite MnO_2 has a layered structure assembled from thin layered MnO_2 nanosheets. After exfoliation during ultrasonication, thin layered MnO_2 nanosheets can be obtained from the solution. **c,** Zoomed-out TEM images of MnO_2 nanosheets that assembled into a porous film. **d,** MnO_2 nanosheet structure created by DFT simulation. The simulated crystal lattice matched with experimental observations in HRTEM analysis. Blue refers to manganese atom, and red refers to oxygen. **e-i,** Zeta size, UV-Visible spectrum, XPS, Zeta potential, TEM sizes, of MnO_2 nanosheets as-synthesized. The highly negative zeta potential around -30 mV could be originated from the hydroxyl groups existent in the defects of MnO_2 nanosheets. This high value of negative zeta potential also indicates good colloidal stability of MnO_2 nanosheets in solution. UV-Vis absorption spectrum of MnO_2 nanosheets shows the

identical absorption of MnO_2 nanosheets in solution at 385 nm, which was used for quantifying remaining MnO_2 in biodegradation studies.

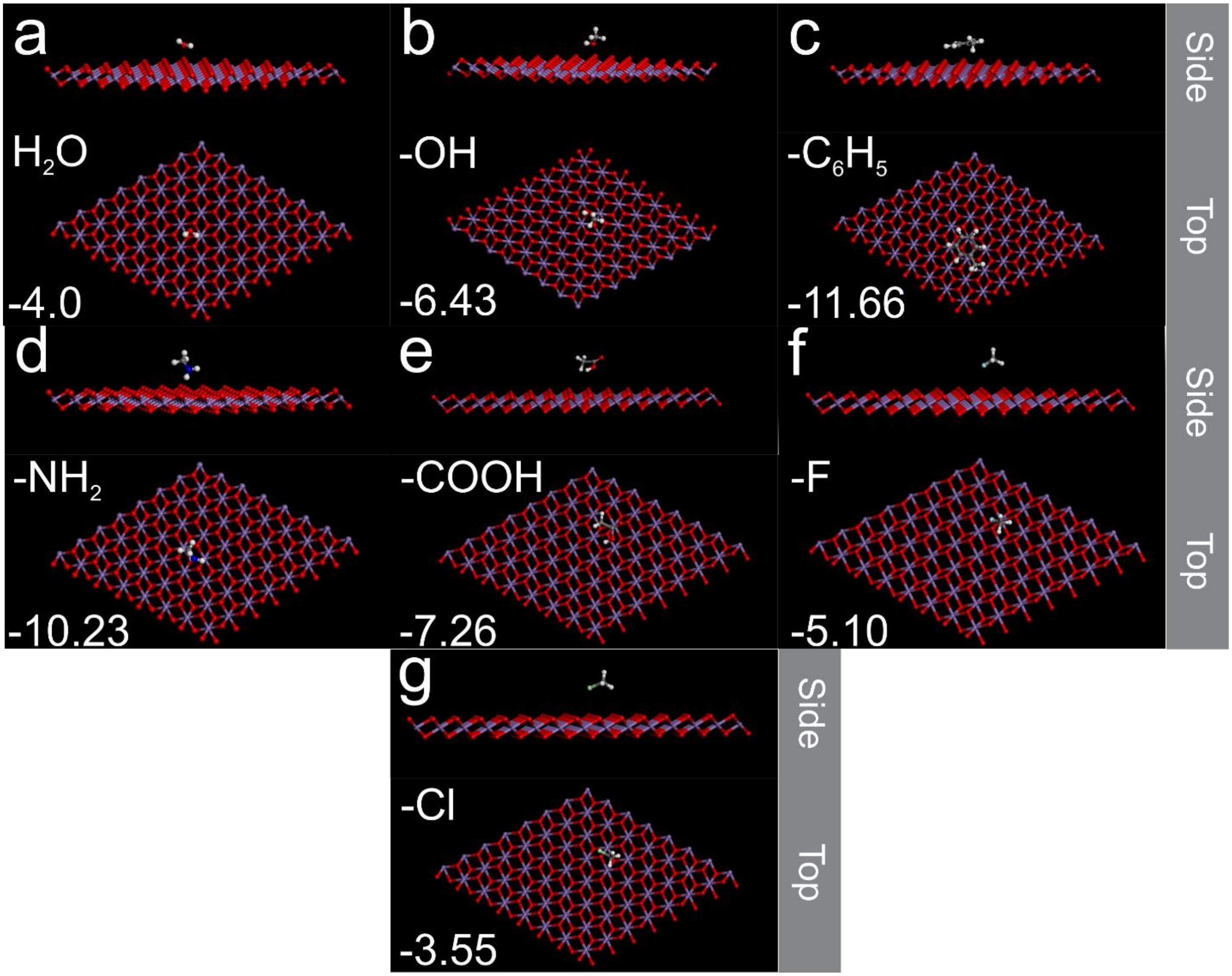

Supplementary Figure 2. Modeling nanosheet-small molecule interactions using DFT calculation. a-g, Computational modeling result describing the optimized geometry (both top views and side views) of binding between MnO_2 nanosheets and water (a), hydroxyl group (b, hydrogen bonding), aromatic benzene ring (c, metal-π polarization), amine group (d, electrostatic interaction), carboxylic group (e), fluoride (f) and chloride (g). The binding energies were calculated by the equation of E(BE)=E(A+B)-E(A)-E(B), and summarized in the left bottom of each figure (BE: Binding Energy; A: Single layer of MnO_2 nanosheet; B: Small molecules with assigned functional groups; Units: kCal/mol). In these simulation models, manganese atom is colored with blue, oxygen atom is colored with red, carbon atom is colored gray, hydrogen is colored with white.

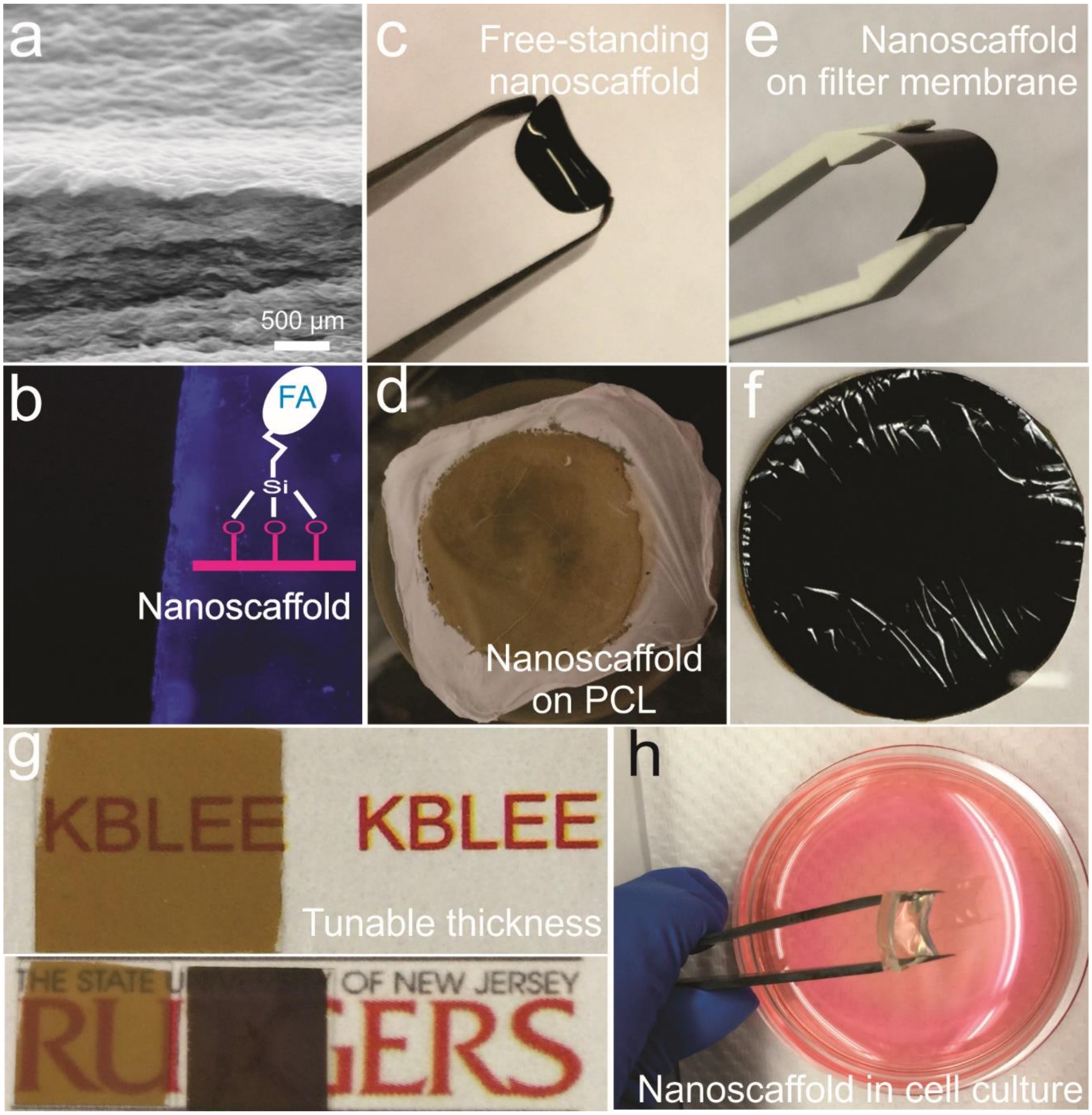

Supplementary Figure 3. Characterizations of MnO_2 hybrid nanoscaffold fabricated by a vacuum filtration method. **a,** A zoomed-in SEM image of nanoscaffold from the side view showing the layered structure of MnO_2 nanoscaffold. **b,** Surface functionality of nanoscaffold. The existence of surface hydroxyl groups on MnO_2 nanosheet enabled the covalent functionality of the nanoscaffold By using APTES. Fluorescence from fluoresceinamine (FA, blue) conjugation was used to visualize the success of conjugation. **c,** Photograph showing a free-standing MnO_2 nanoscaffold. Nanoscaffold fabricated from vacuum filtration is mechanically robust and can be manipulated and held by tweezers. No cracks were found on nanoscaffold during manipulation. **d-e,** A nanoscaffold placed on top of a biodegradable polymer (PCL nanofiber) or cellulose nano-porous membrane. The incorporation of polymer substrate can increase the flexibility of nanoscaffold during cell transplantation. **f,** A photograph showing MnO_2 hybrid nanoscaffold with the homogeneous surface in the large area. The size of the photograph is 25mmx25 mm. **g**, MnO_2 hybrid nanoscaffold with tunable thickness showing varying transparency. This is achieved by adjusting the concentrations of MnO_2 nanosheet solution used for vacuum filtration. **h,** A photograph showing MnO_2 hybrid nanoscaffold on PDMS seeded with cells.

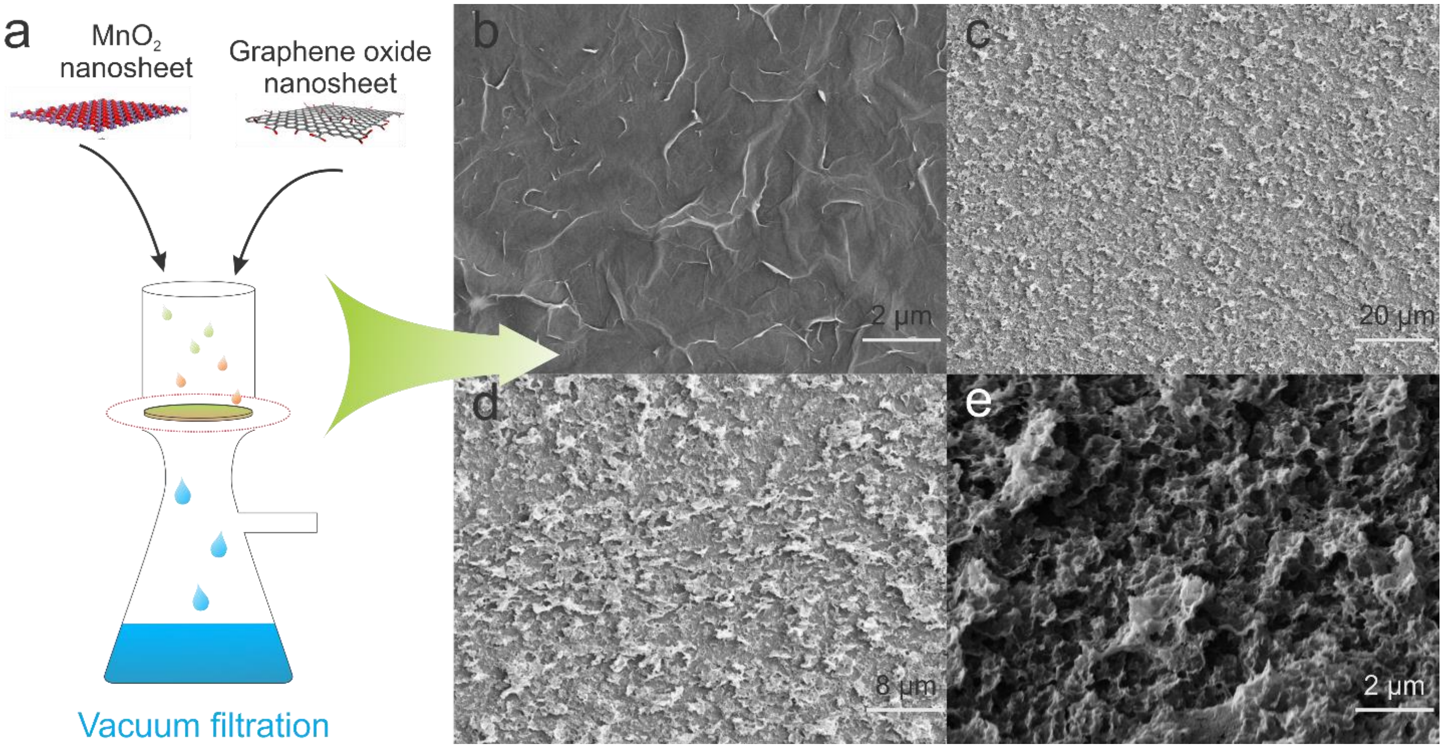

Supplementary Figure 4. Vacuum filtration for fabricating MnO_2 and GO hybrid nanoscaffold. a, Schematic diagram for explaining the vacuum filtration approach for fabricating homogeneous GO nanoscaffold and MnO_2 hybrid nanoscaffold (**a**). **b-e**, FESEM of GO nanoscaffold (**b**) and MnO_2 nanoscaffold (**c-e**). Both nanoscaffolds were fabricated from vacuum filtration method and are highly homogeneous in the large scale. However, the detailed nanotopographies of GO nanoscaffold and MnO_2 nanoscaffold are different. Whereas GO nanoscaffold has a relative smooth surface, MnO_2 nanoscaffold has a much rougher surface with many ripple structures. This difference on substrate nanotopography could be originated from the more rigid bonding of Mn-O-Mn compared on nanoscaffold. Increased surface roughness could be one of the reason for nanoscaffold in promoting adhesion and differentiation of neural stem cells.

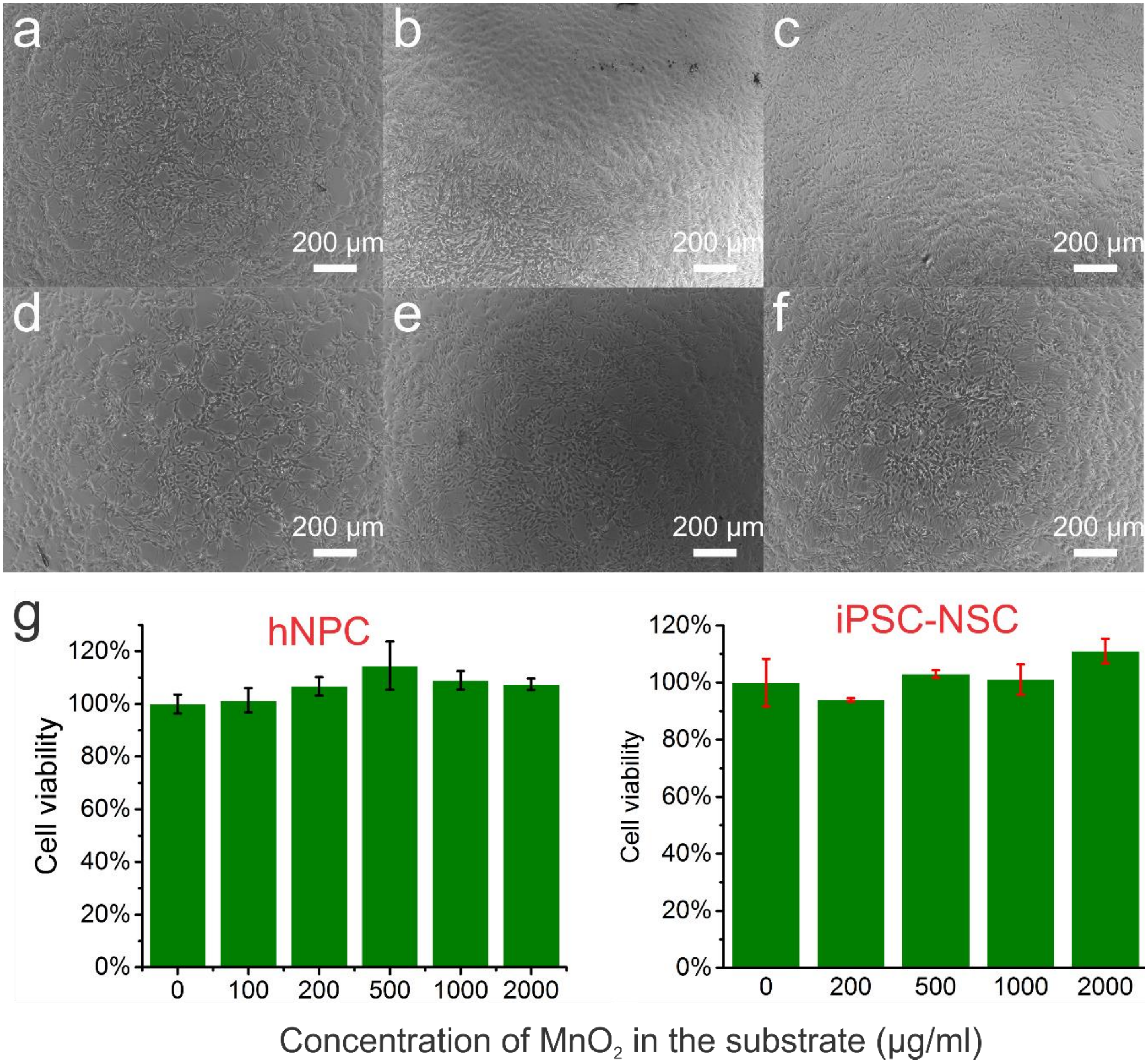

Supplementary Figure 5. Biocompatibility of MnO_2 hybrid nanoscaffold. a-f, Representative images of NSCs adhesion and proliferation on different substrates (**a,d**: glass; **b, e**: graphene scaffold; **c, f**: MnO_2 nanoscaffold) one day after seeding. **g,** Viabilities of cells cultured on glass coated with laminin were used as controls. Cells were cultured on a laminin-coated glass substrate and nanoscaffold for 2 days, and then presto-blue assay was used for quantifying relative cell viability based on the absorption at 570 nm in plate-reader. The high cell viability of both hNPCs and iPSC-NSCs cultured on nanoscaffold indicates its excellent biocompatibilities. Due to the stronger protein absorption on graphene oxide assembled scaffold and nanoscaffold, higher numbers of NSCs were adhered and grown on graphene oxide assembled scaffold and nanoscaffold compared to glass substrates. Data represents mean$\pm$s.d., n=3, results are non-significant (*P>0.05) between different conditions by one-way ANOVA.

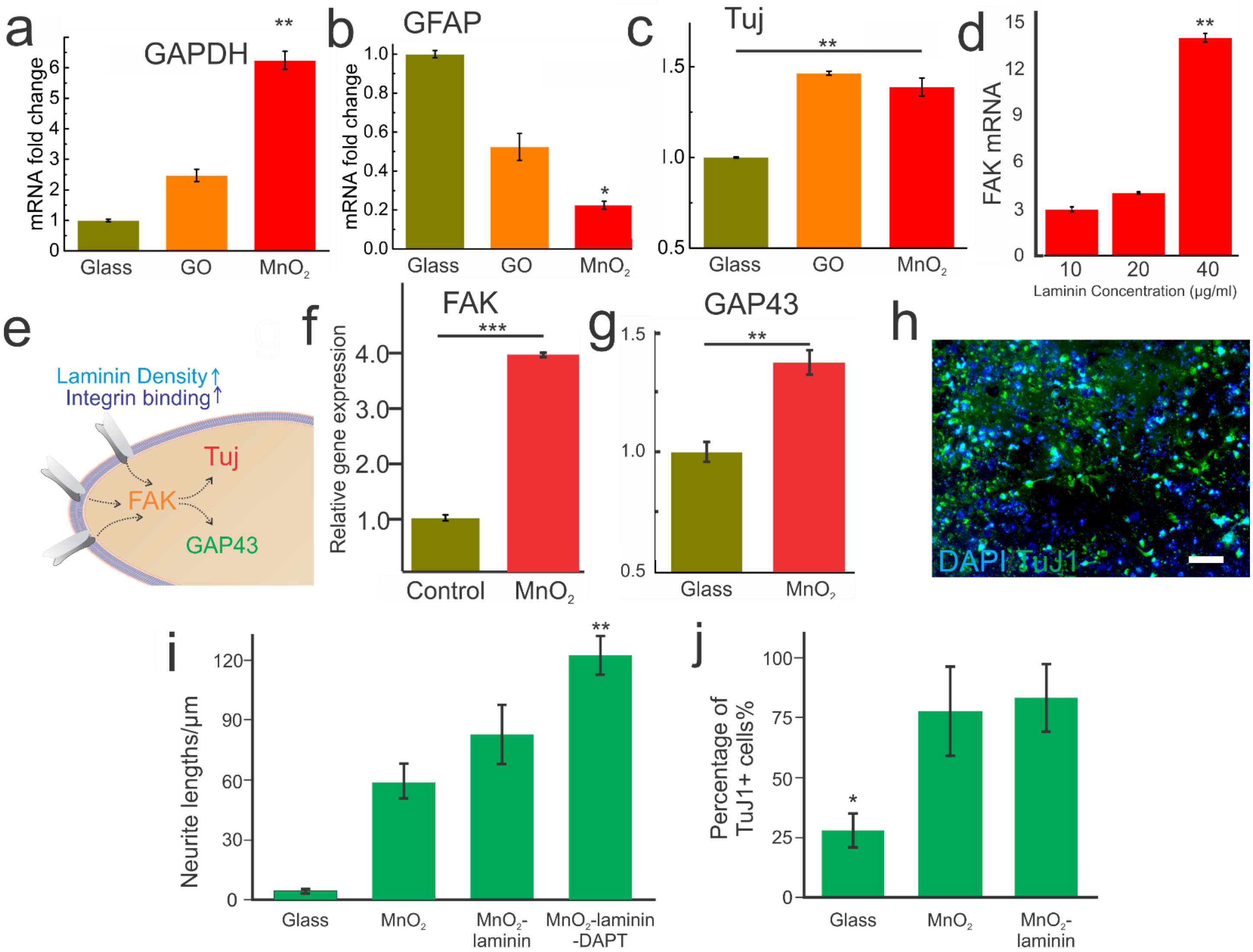

Supplementary Figure 6. Enhanced Neuronal differentiation of iPSC-NSCs on MnO_2 hybrid nanoscaffold. a-g, mRNA levels of GAPDH (**a**), GFAP (astrocyte marker gene, **b**), TuJ1 (early neuron marker gene, **c**), laminin concentration dependent and substrate dependent FAK expression (5 μg/ml, 10 μg/ml, 20 μg/ml, from left to right) (**d, f**) and GAP43 (growth cone associated protein, **g**). **e** is a schematic diagram for proposed pathways summarizing the gene analysis. and comparison of axonal length for iPSC-NSCs differentiated on the different substrate for 6 days. This gene analysis result describes an obvious enhancement of neuronal differentiation on MnO_2 nanoscaffold compared to the control substrate, with a slight suppression of gliogenesis. **h,** Immunostaining of neuronal markers (green) and nuclei staining (blue) for iPSC-NSCs differentiated on GO nanoscaffold, which was used as a positive control comparing to MnO_2 nanoscaffold and the glass substrate in the main Figure 1. **i-j,** Quantified neurite growth of neurons (i) and percentage of neuronal cells. Scale bar: 100 μm (**j**) differentiated from iPSC-NSCs cultured on a different substrate (glass, MnO_2 nanoscaffold, MnO_2 laminin nanoscaffold and 3D BHI nanoscaffold with DAPT loaded). Data are mean±s.d., n=3 for **a-d** and **j**; n=9-12 for **i**, **P<0.01 and ***P<0.001 by unpaired student t-test for **f** and **g**, *P<0.05 and **P<0.01 by one-way ANOVA with Tukey post-hoc analysis for **a-d** and **i-j**.

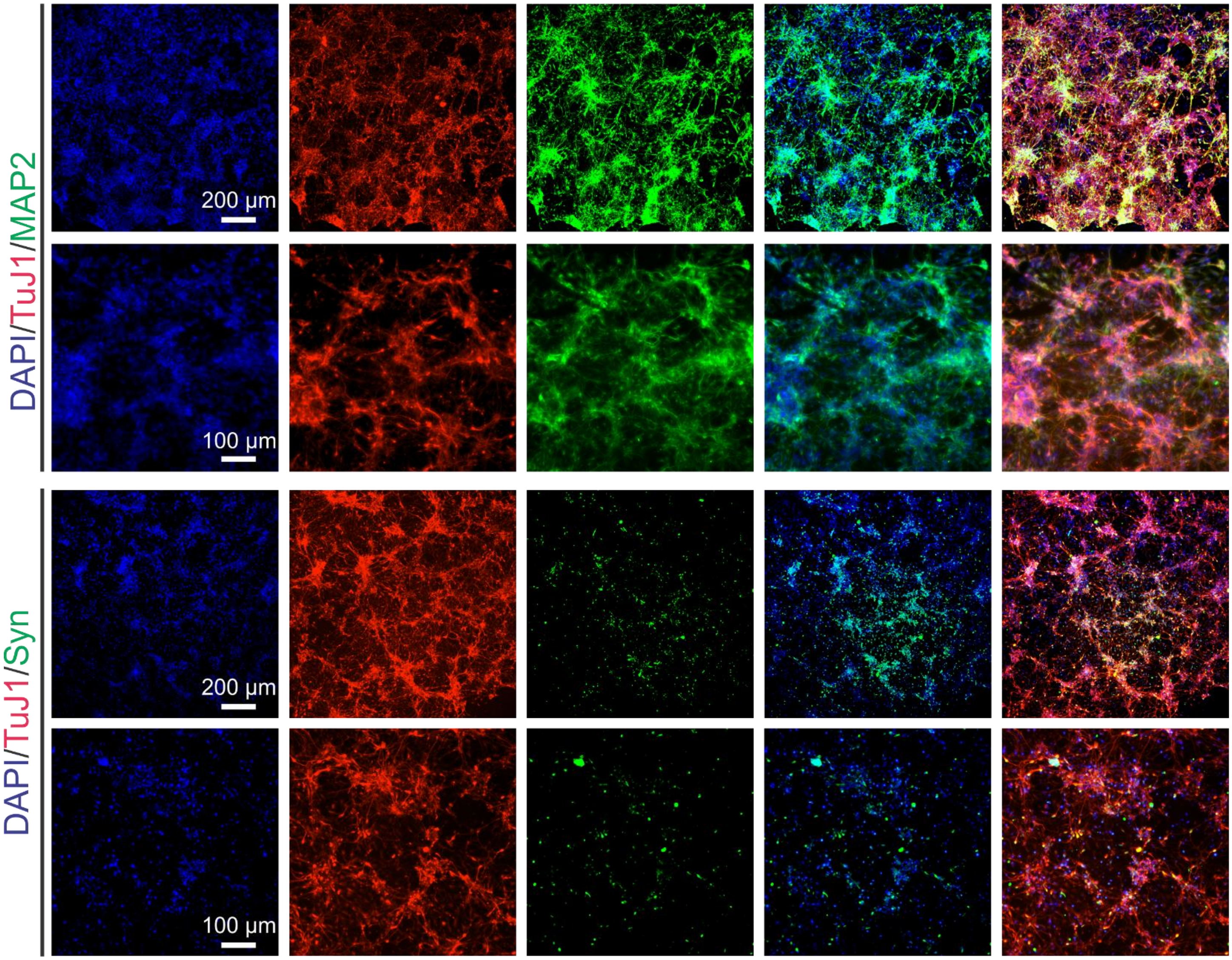

Supplementary Figure 7. iPSC-NSCs differentiated on MnO_2 laminin nanoscaffolds expresses mature neuronal markers (MAP2 and Synapsin 1 (Syn)). Shown in this figure is immunostaining images of iPSC-NSCs differentiated after 6 days.

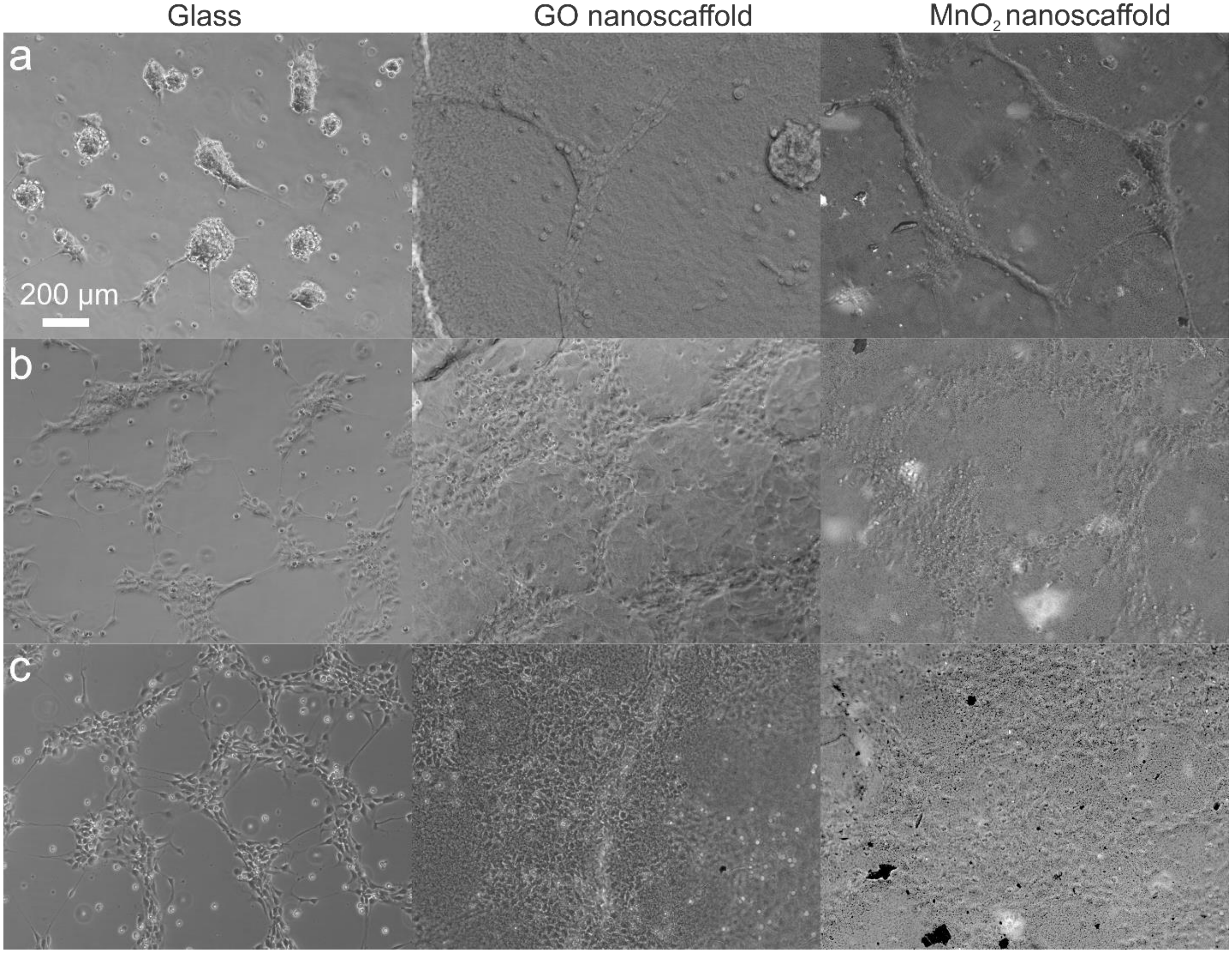

Supplementary Figure 8. Laminin concentration-dependent adhesion of iPSC-NSCs in growth media on different scaffolds. a-c, Phase images of iPSC-NSCs cultured on a different substrate (glass, graphene oxide assembled scaffold, and nanoscaffold) under increasing concentrations of laminin (**a**: 5 µg/ml; **b**: 10 µg/ml; **c**: 20 µg/ml) during coating process. Varying laminin coating concentrations was found to significantly influence the adhesion of iPSC-NSC for all the 3 substrates. However, under low laminin coating concentrations (0.5x and 1x), cells seeded on MnO_2 nanoscaffold and GO nanoscaffold showed obviously improved cell spreading and adhesion. This enhanced adhesion on MnO_2 nanoscaffold and GO nanoscaffold was attributed to increased protein deposition on the surface of the substrates.

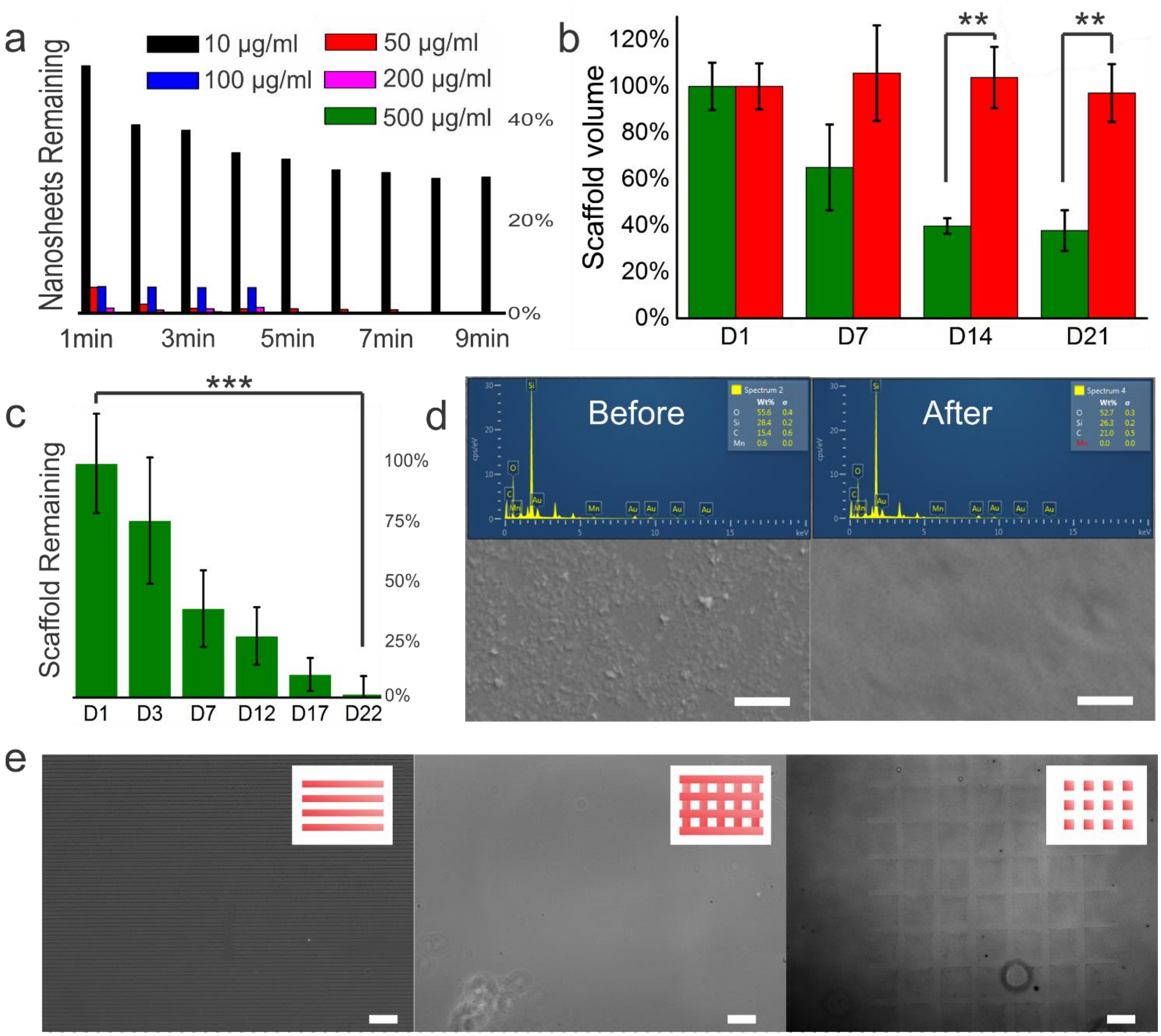

Supplementary Figure 9. Tunable biodegradability of MnO_2 nanoscaffold through an unconventional redox-mechanism. a, Controlled biodegradation of 2D MnO_2 nanosheets by vitamin C in a dosage-dependent manner in physiological conditions. **b,** In the structure of thick layered (6 mm) nanoscaffold sandwiched by two cell layers, nanoscaffold has a half-degradation time around 2 weeks. This is achieved under regular cell culture conditions without the addition of any exogenous biochemical or vitamin C. Green bars (left bars) indicate MnO_2 condition and red bars (bars on the right) indicate GO condition. Data represents mean$\pm$s.d., n=3, *P<0.05 and **P<0.01 by unpaired student t test. c**,** When the thickness of nanoscaffold was significantly reduced to a thin film (<1 mm), the half-degradation time was reduced to less than one week. Data represents mean$\pm$s.d., n=3, ***P<0.001 by one-way ANOVA with Tukey post-hoc analysis. **d,** The successful degradation of nanoscaffold thin film was confirmed with SEM and EDX based elemental analysis. **e,** Micropatterned nanoscaffold assembled from MnO_2 nanosheets using µ-contact printing. Based on the hydrophilic surfaces, nanoscaffold micropatterns with different shapes (line pattern, grid pattern and square pattern) were fabricated using micro-contact printing. These micropatterns were used to visualize the thin nanosheets film as a substrate, and for monitoring the degradation of the scaffold under an optical microscope. In addition, they can also be

used as a substrate for controlling cellular geometries for controlled cell adhesion. Scale bars: **d,** 200 μm; **e,** 100 μm.

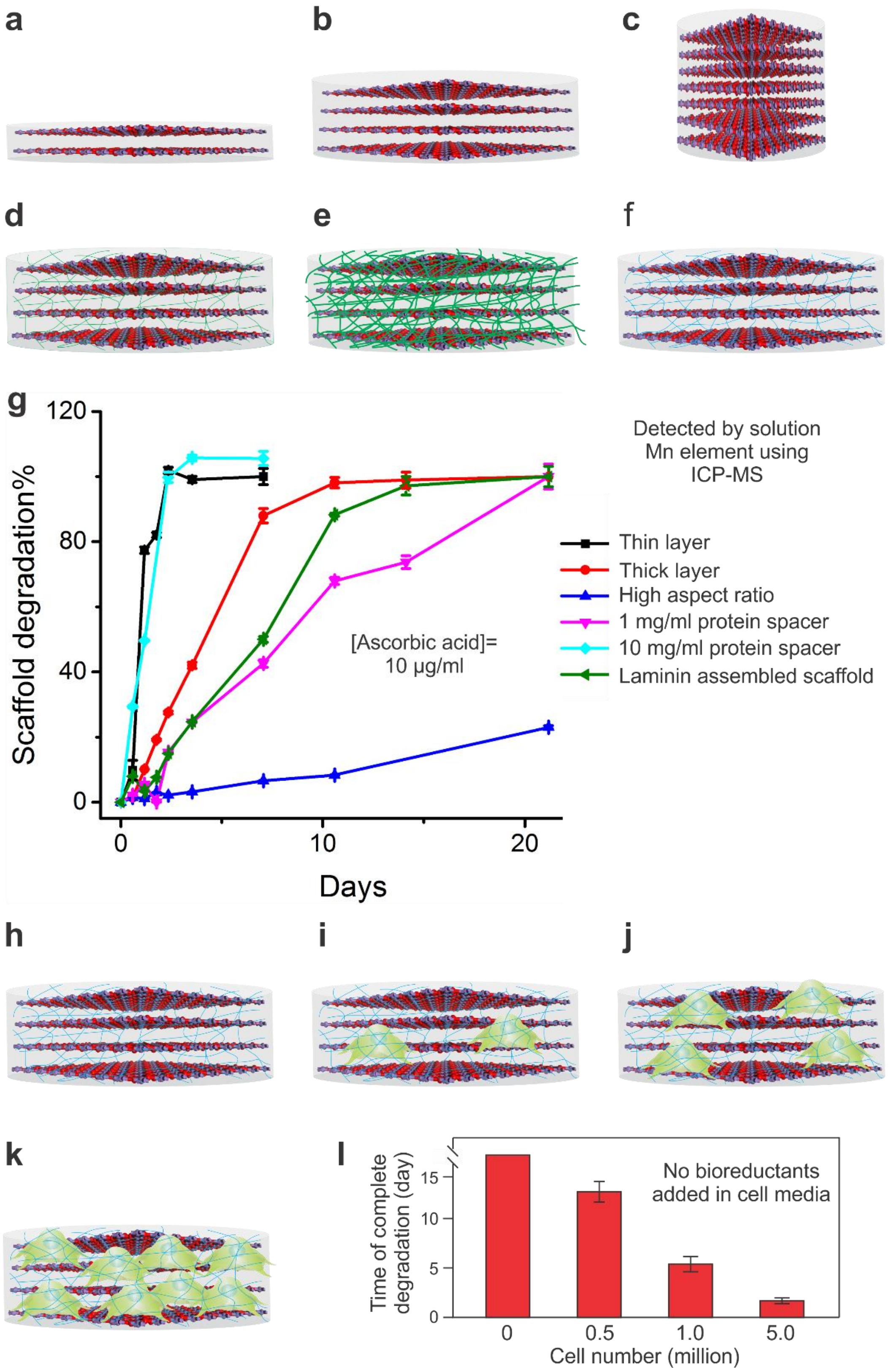

Supplementary Figure 10. Tunable biodegradability of MnO_2 nanoscaffolds by modulating scaffold structure and varying cell densities. a-f, 6 different methods to prepare scaffolds and for modulating scaffold degradation. Symbols were kept consistent with Scheme in manuscript. Green fibers in d and e represent bovine serum protein. **b** represents the control scaffold to show the tuning of biodegradability by comparing it to **a**, and **c-f**. g, Degradation profile of different scaffolds obtained from measuring time-dependent manganese concentrations in the solution using ICP-MS. **h-l**, Tuning scaffold degradation by controlling cell amount transplanted on the scaffold. **h-k**, Schematics showing scaffolds seeded with varying amount of iPSC-NSCs. **l**, A summary of full degradation time of scaffolds based on the complete disappearance of scaffold color. MnO_2 laminin nanoscaffold was used for all the conditions, and regular iPSC-NSC differentiation media was used for maintaining cell viabilities with regular daily change. n=3, 2 in g and m, respectively. Error bars are standard error of the mean.

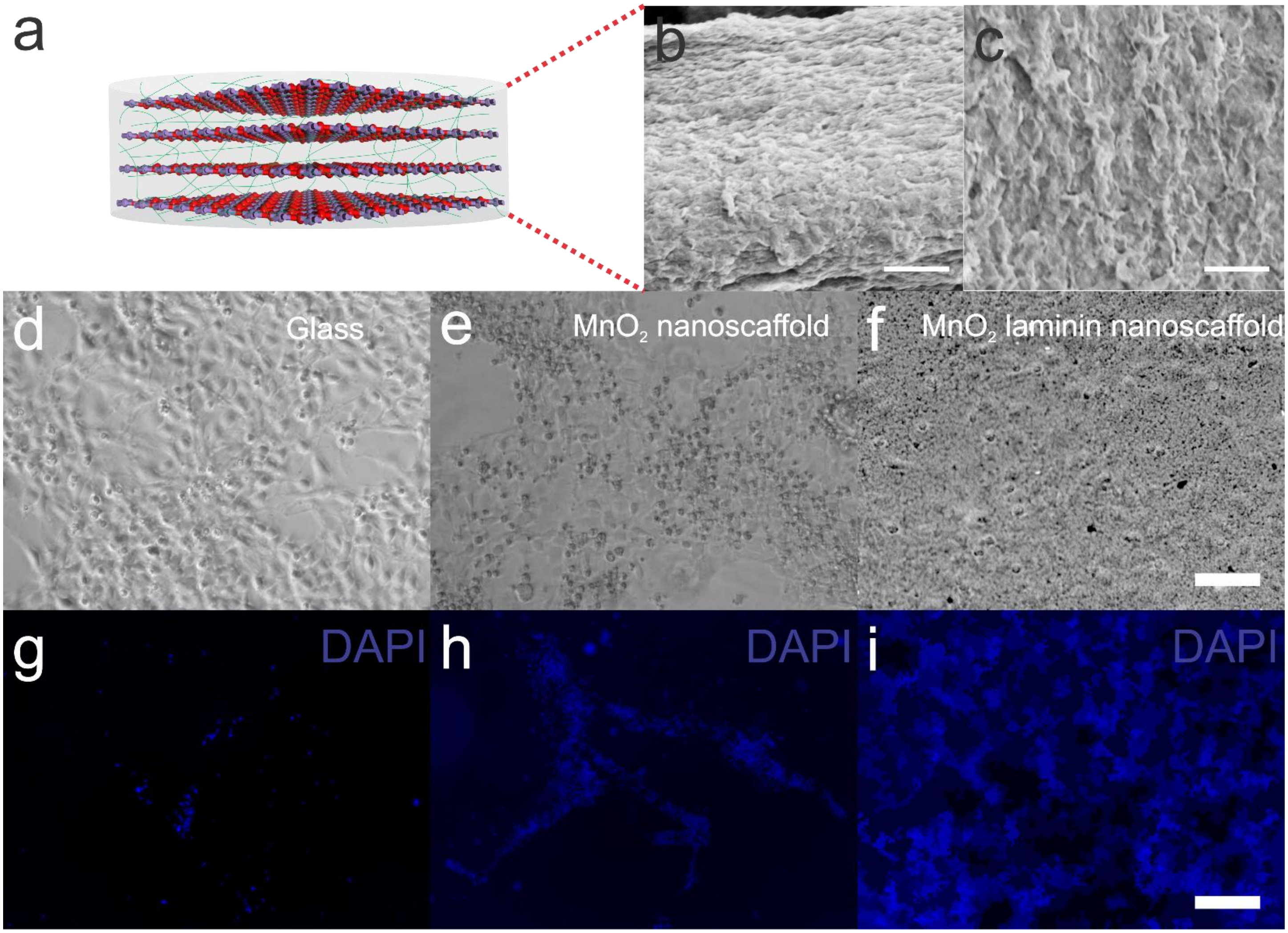

Supplementary Figure 11. Cellular adhesion of iPSC-NSCs on MnO_2 laminin hybrid nanoscaffold during 6 days' differentiation of iPSC-NSC. a, Schematic diagram of MnO_2 nanosheet assembled into laminin-nanoscaffold. **b,** FESEM images of MnO_2 laminin hybrid nanoscaffold indicate it has a highly rough surface and the 2D MnO_2 nanosheet was spontaneously assembled into bulk substrates. **d-f,** Phase images of cells cultured on glass coated with laminin (**d**), nanoscaffold coated with laminin (**e**), and laminin-nanoscaffold (**f**) for one day. From the phase images, cell densities are similar between these 3 after seeding. **g-i,** Nuclei staining (blue) on cell adhesions of iPSC-NSCs differentiated for 6 days on glass coated with laminin (**g**), nanoscaffold coated with laminin (**h**), and laminin-nanoscaffold (**i**). These images clearly show that laminin-nanoscaffold significantly improved adhesion of iPSC-NSCs over the 6 days' differentiation, due to its uniformly distributed laminin adhesion proteins. Scale bars: **b**, 1 μm; **c**, 500 nm; **d-f**, 100 μm; **g-i**, 200 μm.

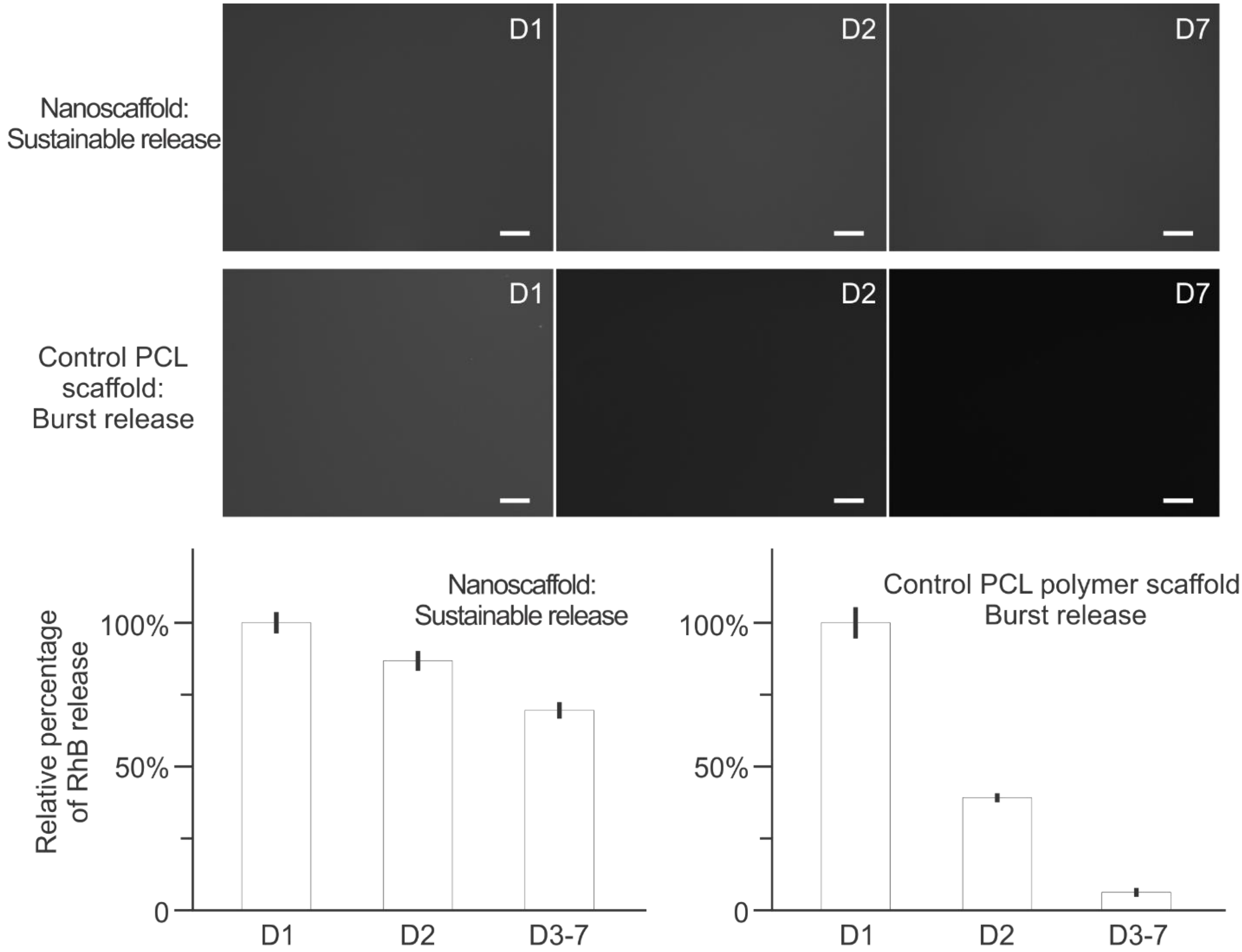

Supplementary Figure 12. Drug (RhB) daily releasing profiles from MnO_2 laminin hybrid nanoscaffold and control polymer (PCL) scaffold. Compared to PCL scaffold, nanoscaffold has a more sustainable release profile through the one week's processes. RhB releasing was indicated by the brightness of monocolored images (top) and summarized in relative percentage compared to the amount of released at Day1.Scale bars: 200 µm. Error bars are the standard error of the mean (n=5).

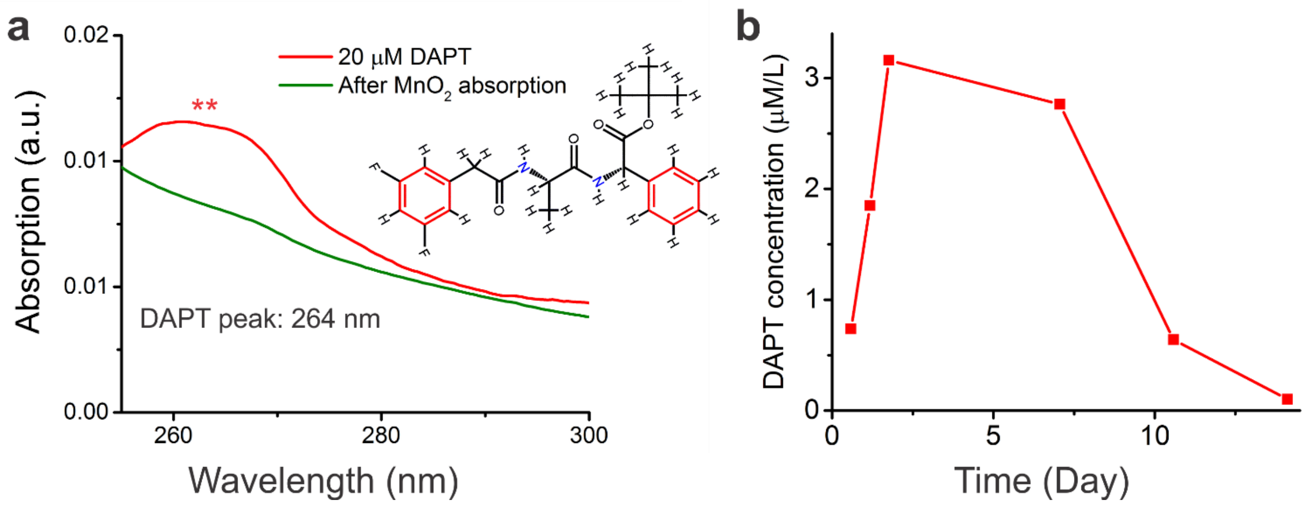

Supplementary Figure 13. Efficient DAPT loading and sustainable release of DAPT on MnO_2 nanoscaffold. a, UV-Vis spectrum of DAPT solution before and after MnO_2 nanosheets absorption. Based on the disappearance of the characteristic peak of DAPT at 264 nm, we can quantify the excellent absorption of DAPT by nanosheets. Inset is a chemical structure of DAPT. **b**, Sustainable DAPT releasing from MnO_2 nanoscaffold. Daily DAPT release was averaged from the total DAPT released over the period of study. Between Day1 and Day10, DAPT concentrations are between 0.6 μM to 3.2 μM, which are within the therapeutic window of DAPT for guiding stem cell neurogenesis.

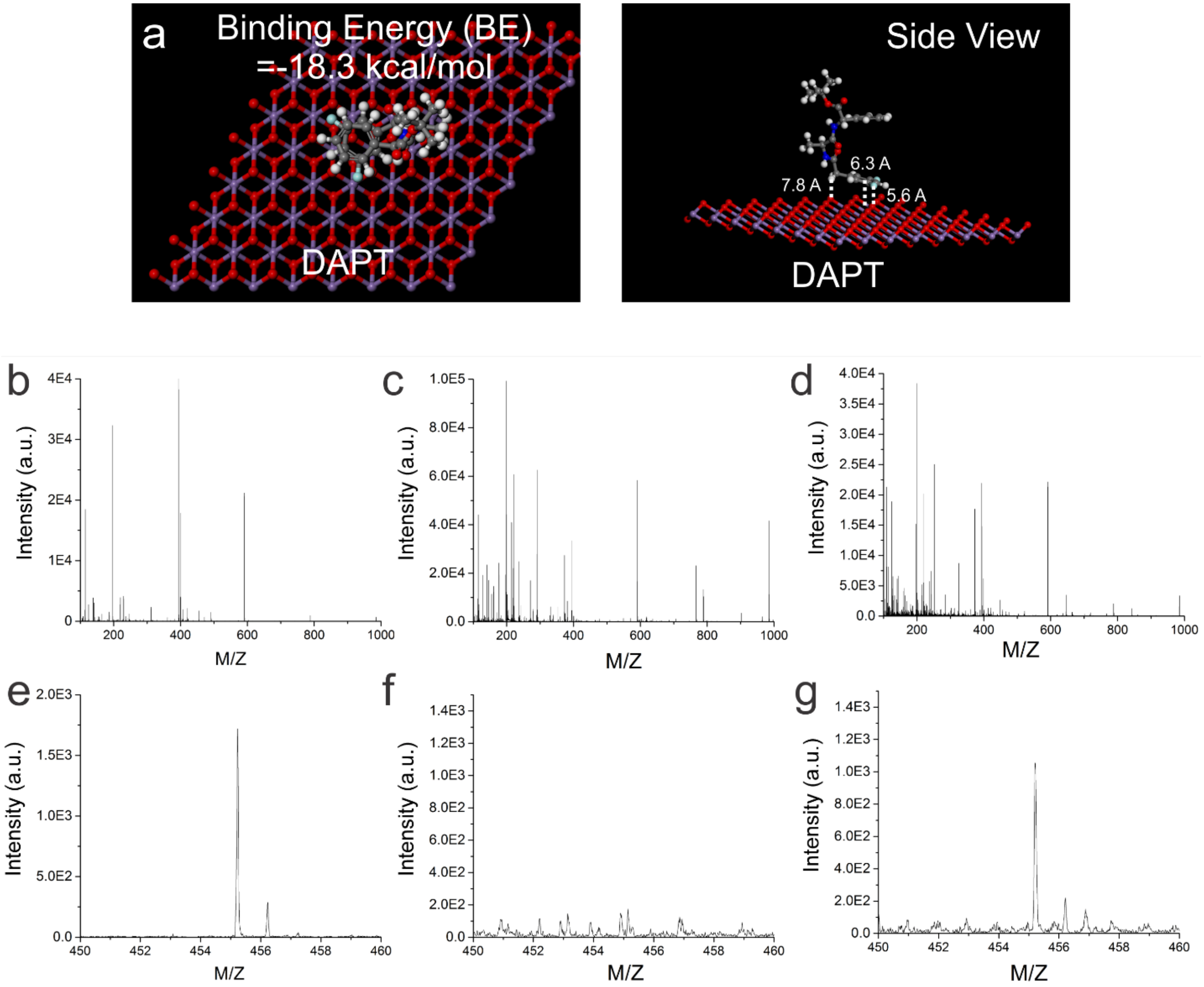

Supplementary Figure 14. Neurogenic drug loading onto MnO_2 laminin hybrid nanoscaffolds. a, Computational modeling of drug-scaffold interactions based on highly precise DFT calculation. Computational modeling result describing the binding between 2D MnO_2 nanosheets and DAPT, a Notch inhibitor that known to selectively guide neural stem cell towards neuronal lineages. The binding energy was calculated by the equation of E(BE)=E(A+B)-E(A)-E(B), and was summarized in the unit of kCal/mol. Among the modeling result, **a** is the top view of optimized geometry; Image on the right is a side view of optimized geometry. The vertical distances of selected atoms to the surface of MnO_2 nanosheet were labeled in **b**. Modeling of MnO_2 binding with neurogenic or anti-inflammatory drugs other than DAPT (e.g., retinoic acid and BET inhibitors) have been conducted, but results were not shown here. **b-g,** MALDI-TOF analysis of Notch inhibitor absorbed on different substrates. **b, e,** 1.0 mg/ml DMSO solution of DAPT deposited on ITO substrate. **c, f,** Polymer substrate (porous cellulose) absorbed with DAPT followed by washing. **d, g,** DAPT-nanoscaffold. DAPT was first loaded on individual MnO_2 nanosheets in PBS solution, then assembled into DAPT-nanoscaffold using vacuum filtration method.

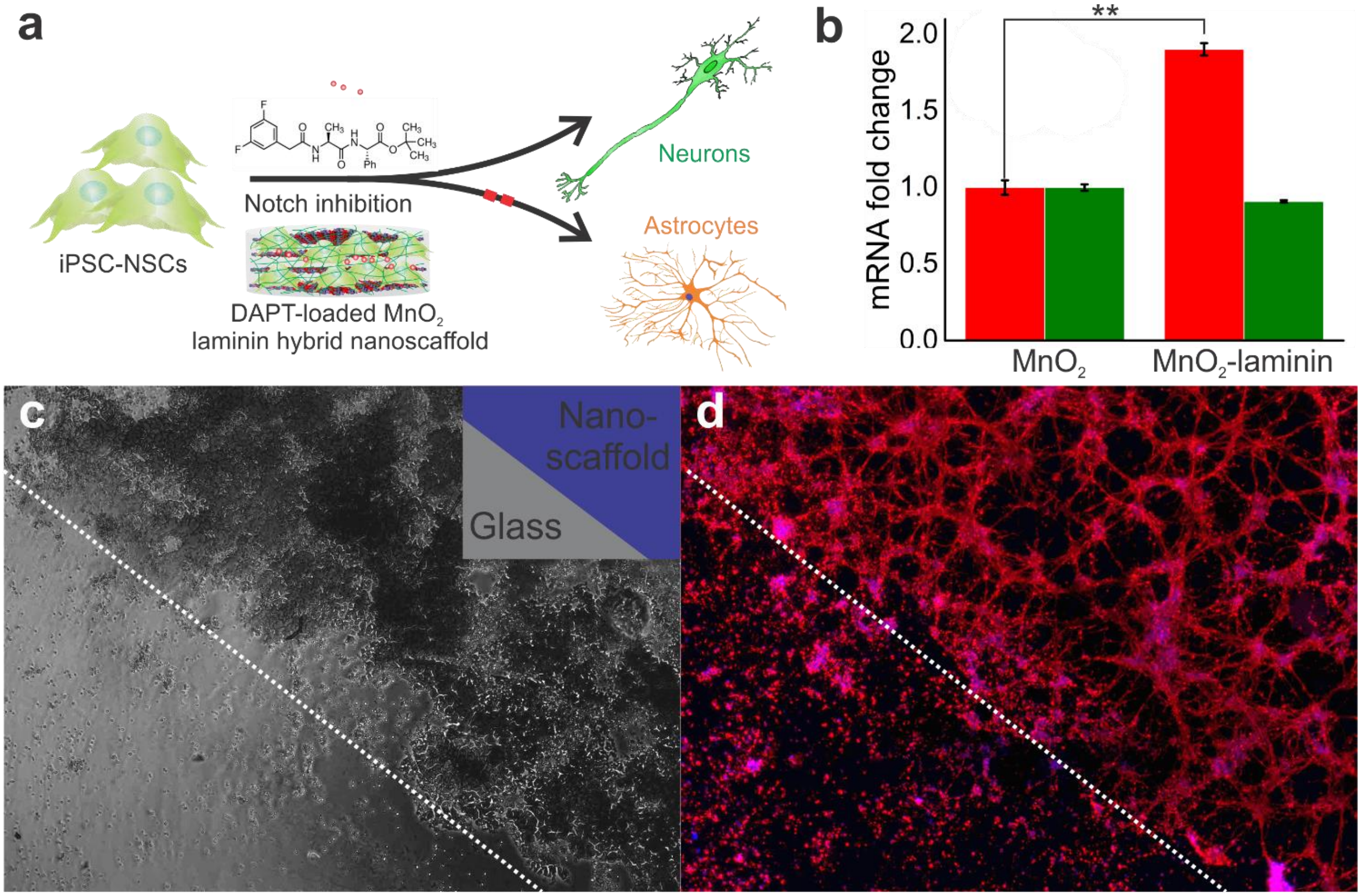

Supplementary Figure 15. Enhanced neuronal differentiation of iPSC-NSCs from DAPT-loaded MnO_2 laminin hybrid nanoscaffold (short-term study). Immunostaining was performed 7-day post transplantation. **a,** Schematic diagram of guided and enhanced neurogenesis of iPSC-NSCs by Notch inhibition. **b,** Summarized gene (TuJ1 and GFAP) analysis and comparison of neurite outgrowth of neurons differentiated from iPSC-NSCs on MnO_2 hybrid nanoscaffold and MnO_2 hybrid. **c-d,** Phase image (**c**) showing the boundary between DAPT-loaded MnO_2 laminin hybrid nanoscaffold (darker regions) and glasses (lighter regions), and their corresponding neuronal and nuclei marker staining (**d**). All the cells are cultured under identical media conditions in the same well of 24-well plate. This image directly proves the enhanced cellular adhesion and differentiation of iPSC-NSCs on DAPT-MnO_2 laminin hybrid nanoscaffold. Scale bars: **c-d,** 250 µm; Data in **b** represents mean$\pm$s.d., n=3, **P<0.01 by unpaired student t-test.

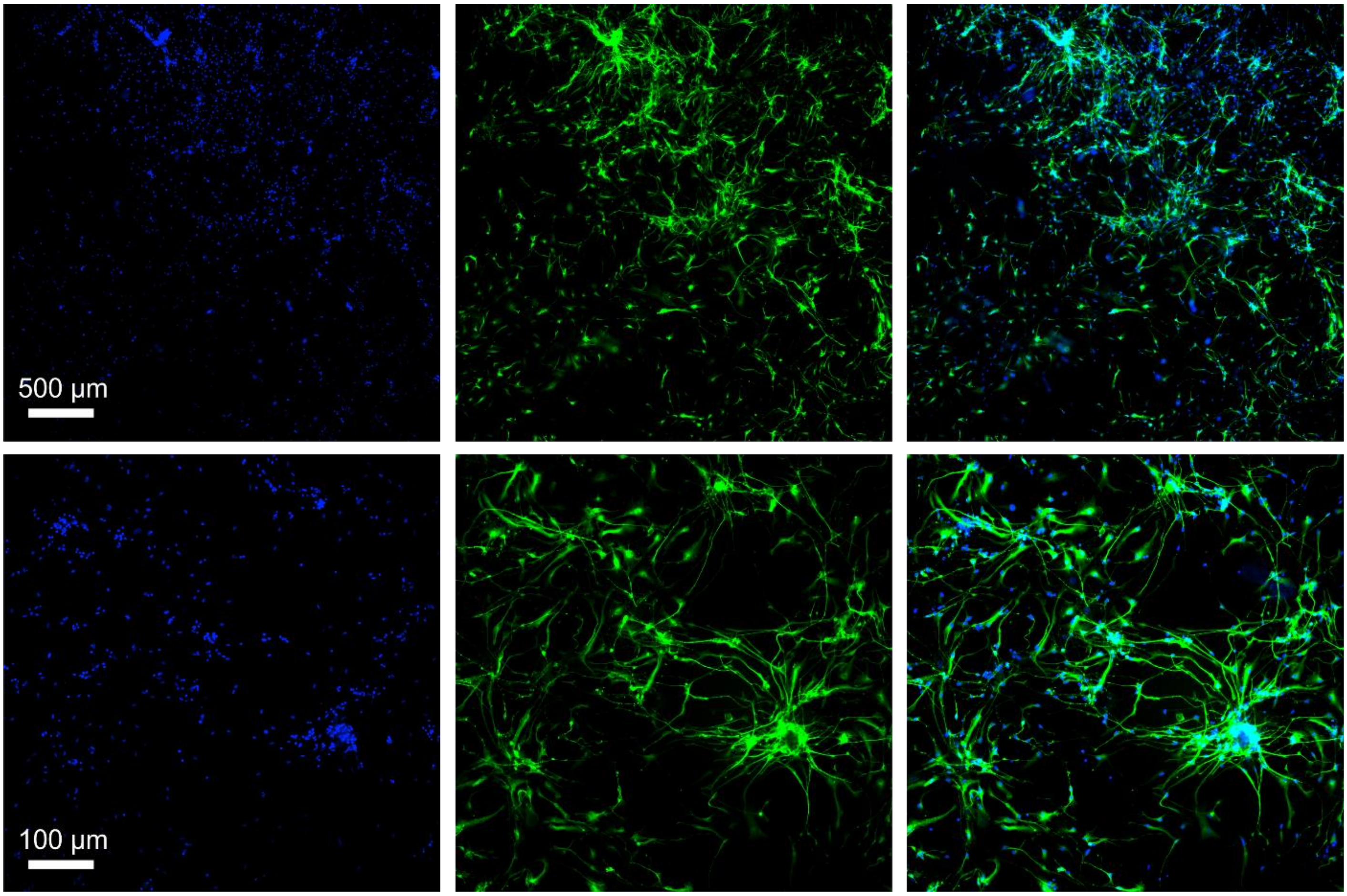

Supplementary Figure 16. Long-term neuronal differentiation of iPSC-NSCs from DAPT-loaded MnO_2 laminin hybrid nanoscaffold. Immunostaining was performed 1 month (30 days) post transplantation. Blue indicates nuclei staining and green indicates mature neuronal marker MAP2. These results clearly support that large populations of differentiated cells and their neuronal fates remained after long-term (1-month) differentiation process on DAPT-loaded MnO_2 laminin hybrid nanoscaffold.

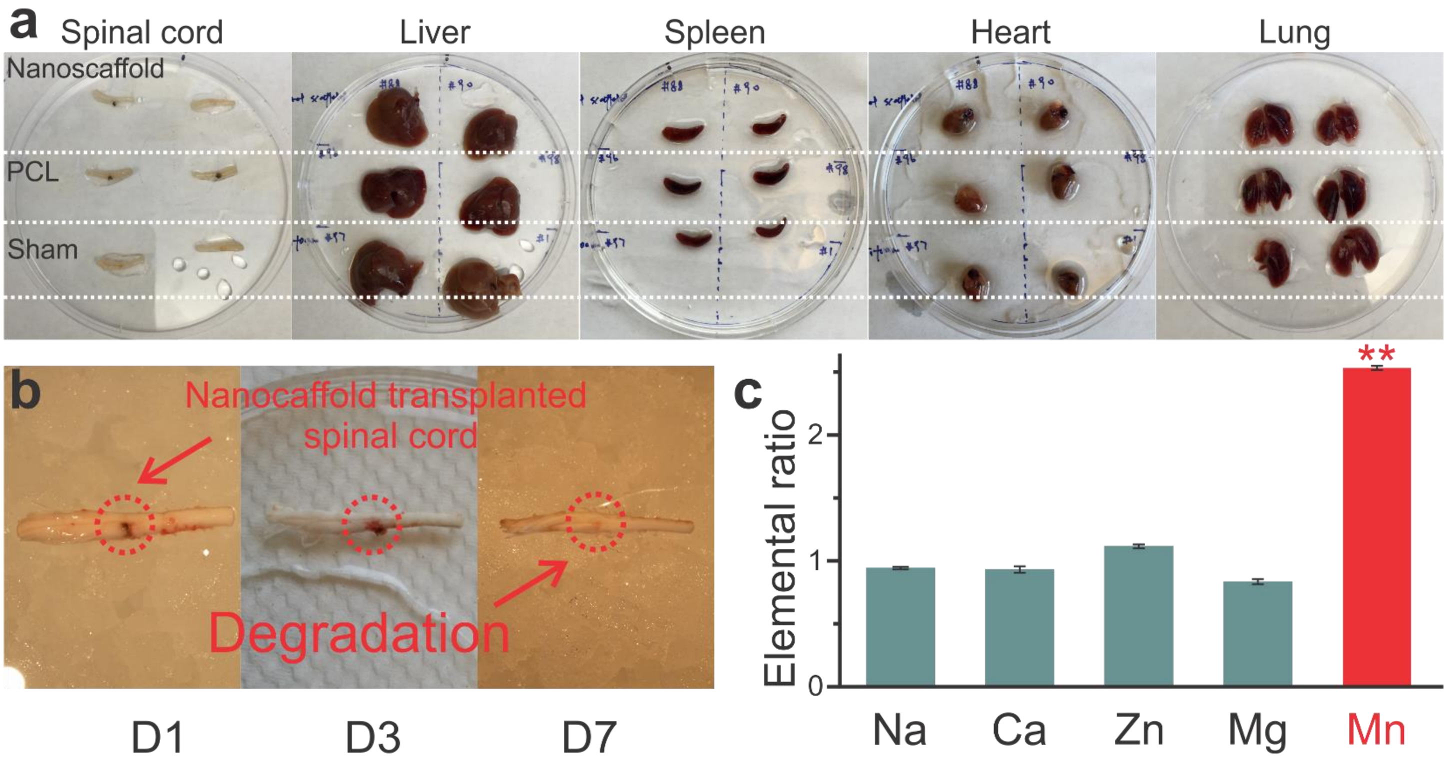

Supplementary Figure 17. ***In vivo*** **Biocompatibility and biodegradability of MnO_2 laminin hybrid nanoscaffolds.** **a,** Photograph comparison on shapes of organs between mice transplanted with iPSC-NSC seeded MnO_2 nanoscaffold (top row), iPSC-NSC seeded polymer scaffold (middle row) and sham with surgifoam (no cells, bottom row). These images demonstrate that no obvious change in organ size, especially in liver and spleen, and minimal *in vivo* toxicity from the transplantation of nanoscaffold and polymer scaffold. **b,** Photographs of nanoscaffold transplanted mouse spinal cord at Day 1, Day 3, and Day 5 from left to right, respectively. These images suggest the time-dependent degradation of nanoscaffold (indicated by the disappearance of black color from nanoscaffolds), as well as a gradual healing of the wound on the hemisected spinal cord. **c,** Elemental analysis on urine samples of mouse 3 days after transplantation of nanoscaffold and polymer scaffolds using ICP-MS. Among the 6 common elements (Ca, Zn, Na, Fe, Mn, Mg) detected in the urine, only Mn level was significantly increased in nanoscaffold compared polymer control in the elemental analysis, which we attribute to the degradation of MnO_2 transplanted in the spinal cord region. Data are mean$\pm$s.d., n=3, *P<0.05 by one-way ANOVA with Tukey post-hoc test.

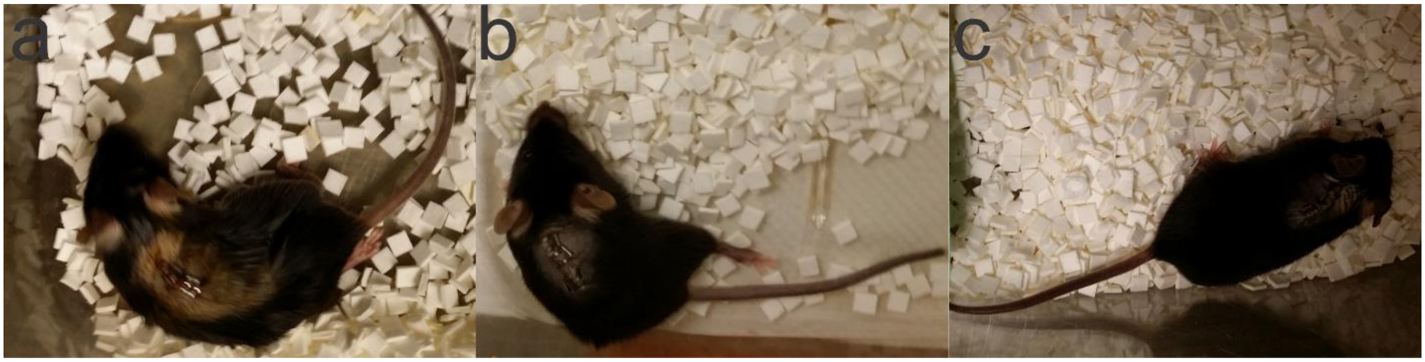

Supplementary Figure 18. Representative photographs from SCI mice without any treatment (a) or transplanted with neural stem cell transplantation using polymer scaffold (b) or nanoscaffold (c) 3-week post injury.

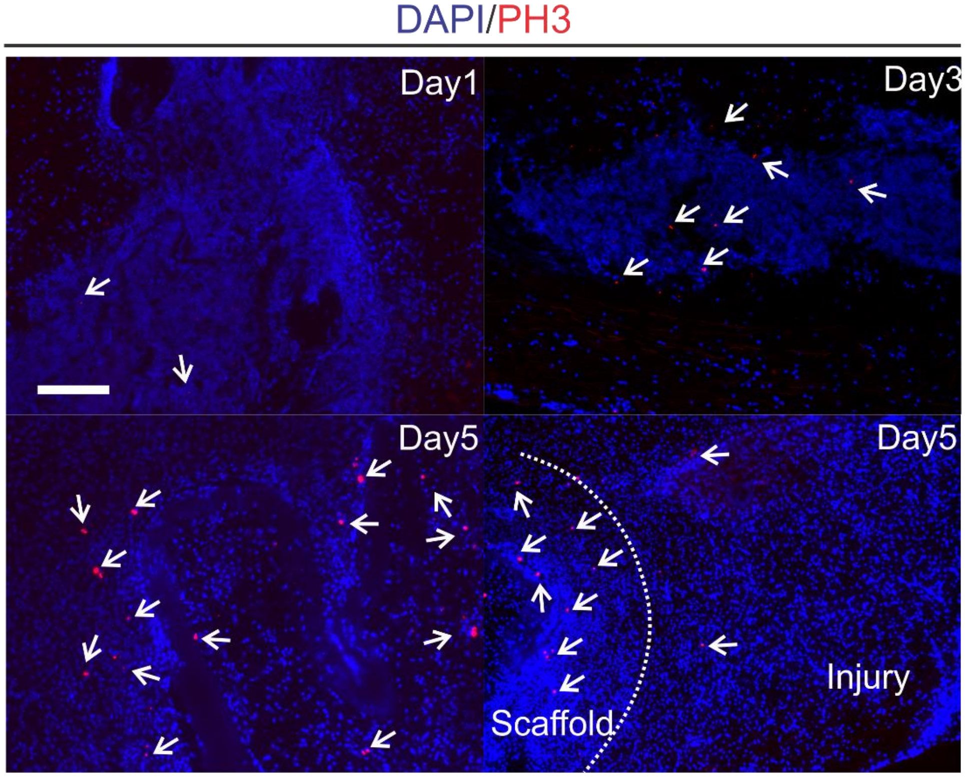

Supplementary Figure 19. Cell proliferation in the MnO_2 laminin hybrid nanoscaffolds nanoscaffolds after transplanted into Notch1CR2 GFP mice. Day dependent (Day 1, Day 3 and Day5) proliferation marker staining for spinal cord injury after transplanted with iPSC-NSC seeded MnO_2 nanoscaffold. With increased days of transplantation, increased proliferation marker (PH3) was found surrounding the scaffold, while there is a minimum amount of PH3 markers found beyond the regions of transplanted scaffold and cells in the injury sites, indicating the growth factor loaded nanoscaffold can promote the survival and proliferation of cells surrounding the scaffold. Scale bar: 100 μm.

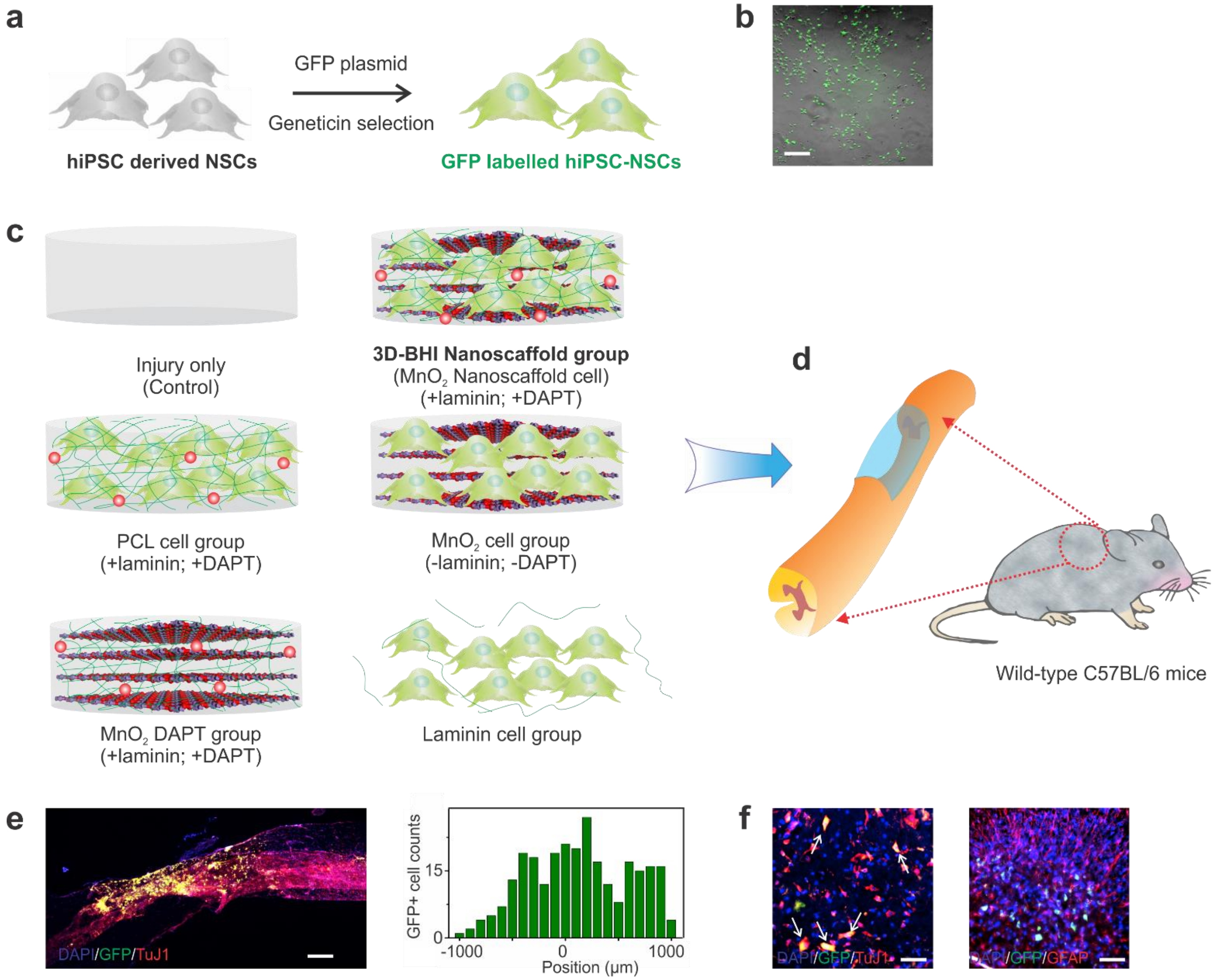

Supplementary Figure 20. Animal groups for studying the effects of 3D-BHI nanoscaffold on the enhanced transplantation of iPSC-NSCs into the SCI sites. All tissue analysis was performed 7-week post transplantation. **a**, Schematic diagram illustrating the plasmid-based transduction and anti-biotics (geneticin) based selection of GFP labelled iPSC-NSCs (GFP-iPSC-NSCs). By utilizing GFP-iPSC-NSCs, transplanted cells can be reliably tracked, and their *in vivo* differentiation can be accurately studied. **b**, Merged fluorescent and phase images of transfected iPSC-NSCs. Through the plasmid transfection and geneticin selection, a high percentage of the iPSC-NSCs (91%, 318 out of 349) showed bright GFP signals. **c-d**, Schematics of 6 experimental groups (**c**) and their transplantation into wild-type C57BL/6 mice (**d**). Indications of symbols in the schematics can be found in Figure 1 of the manuscript. **e**, Zoom-out images showing the distributions of GFP-iPSC-NSCs transplanted by nanoscaffold. The graph on the right is a summary of the distance-dependent distribution of cells from the transplantation site. By utilizing GFP labeled cells, we can not only clearly track the transplanted cells that are diffuse into host spinal cord but also confirm the majority of transplanted cells showed early neuronal markers by identifying the GFP+TuJ1+ cells. **f**, Zoom-in images with DAPI-GFP-TuJ1 (image on the left) and DAPI-GFP-GFAP (image on the right) dual labeling. As shown in the pictures obtained from the nanoscaffold group, we can observe most (6 out of 7) GFP+ cells were TuJ1+, while only minor (1 out of 9 GFP+ cells were GFAP positive, indicating a successful induction of neurogenesis in vivo. More detailed co-labeling experiments and the comparison between nanoscaffold group to other groups will be shown in other Supplementary Figures. Scale bars: **b**, 100 μm; **e**, 250 μm; **f**, 50 μm.

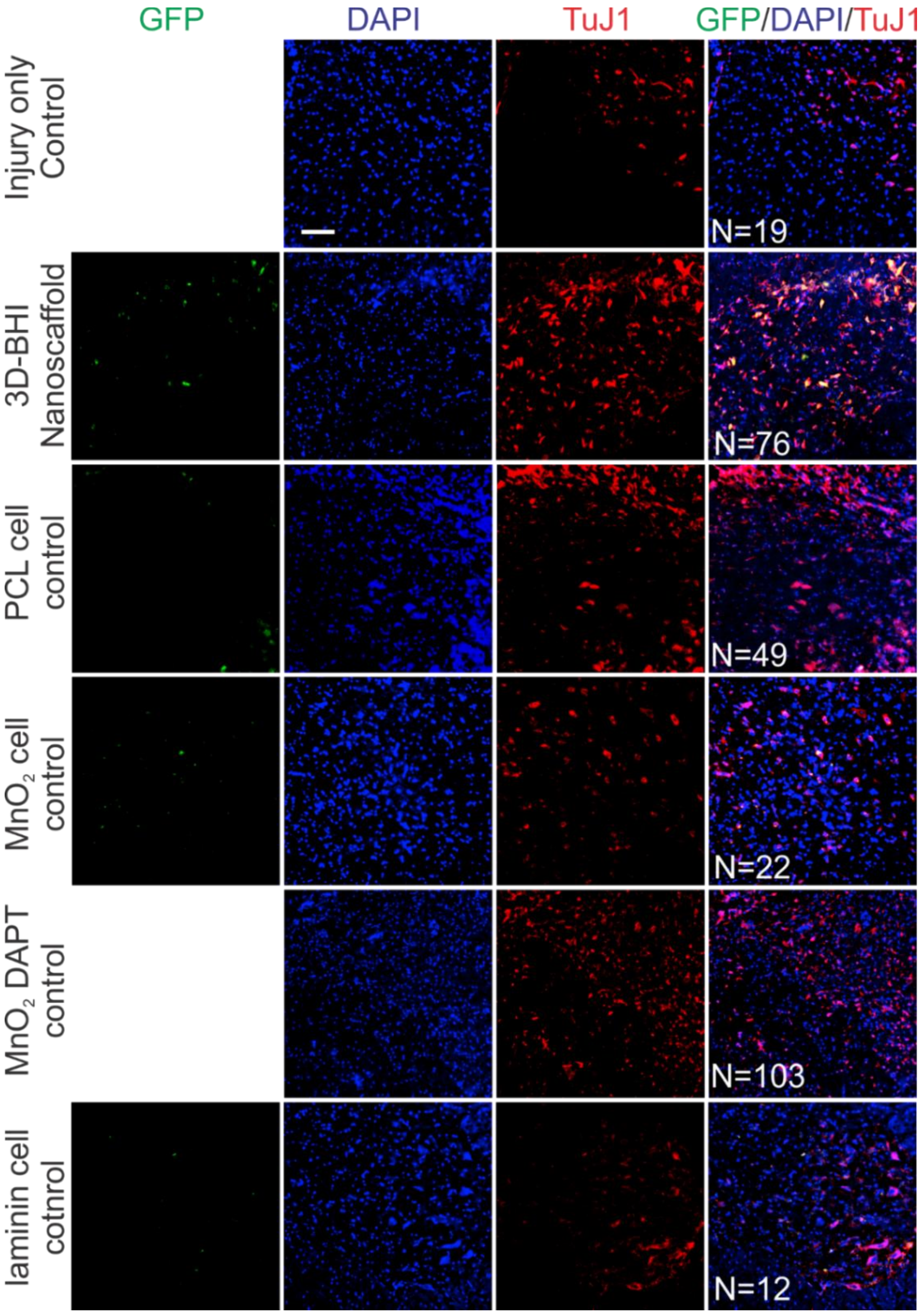

Supplementary Figure 21. 3D-BHI nanoscaffold enhances neuronal differentiation. All tissue analysis was performed 7-week post transplantation and all tissue sections were from the center of spinal cord injury and nearby the transplantation sites. Immunohistological staining was performed to determine the effects on eNSC neurogenesis from different experimental treatments. A high number (N) of eNSC derived neuronal cells was observed in nanoscaffold group and MnO_2 DAPT group. Scale bar: 100 μm.

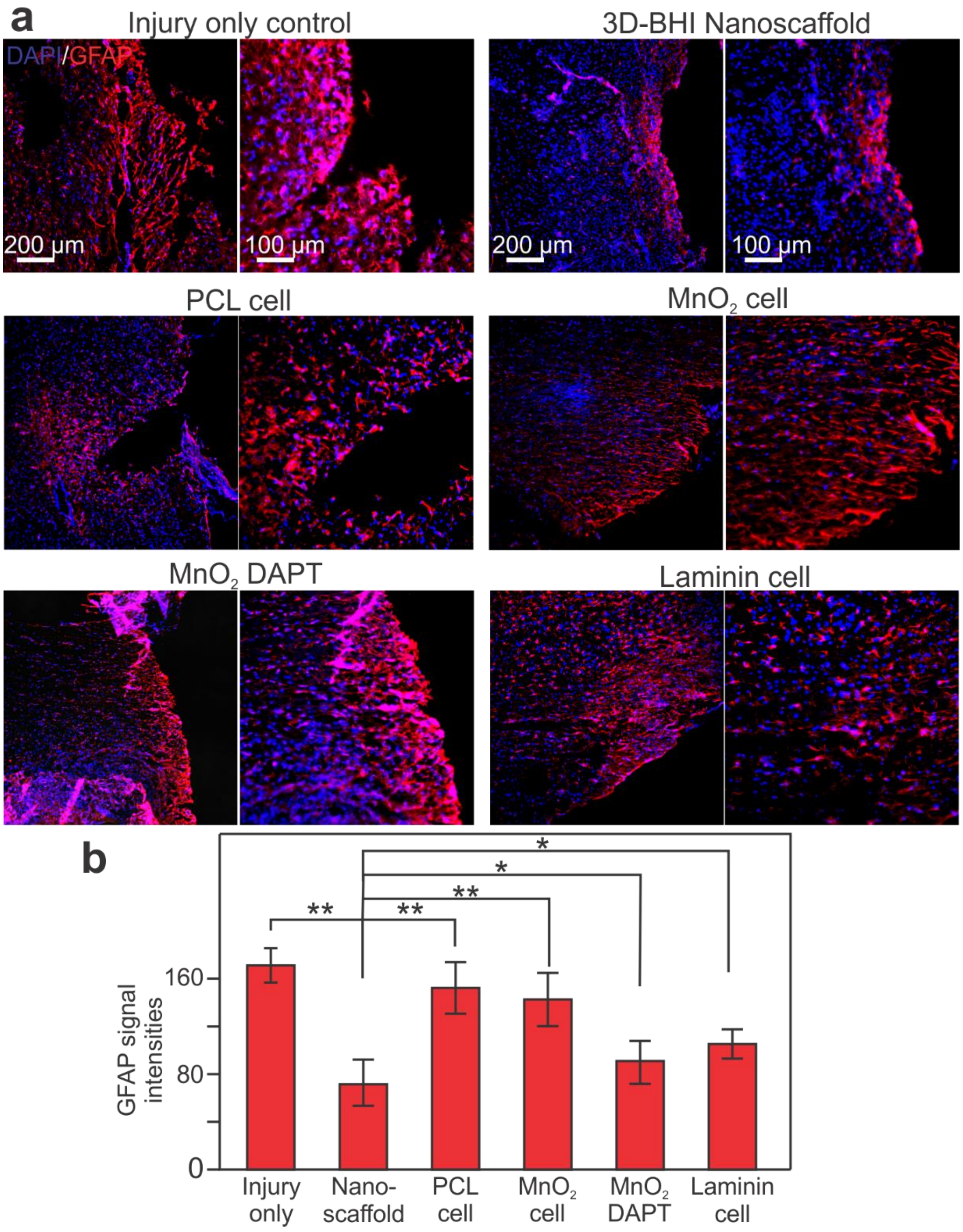

Supplementary Figure 22. MnO_2 nanoscaffold suppresses astroglial activity. Immunohistological staining analysis on the effects of astroglial cell activities at the injured sites was performed 1-week post injury. All tissue sections were from the center of spinal cord injury and nearby the transplantation sites. **a**, Representative images from different groups of scaffold transplantation. Blue represents nucleus staining (DAPI); red indicates astroglial cells (GFAP). **b**, Quantification of GFAP signal intensities (area=132 μm^2, n=3). Exposure time was kept constant at 1 second for all sections from different experimental groups to obtain GFAP signal intensities. Automatic intensity detection modules in the ND2 Nikon software were used for measuring the signals. Data are mean±s.d. n=3, *P<0.05, **P<0.01 by one-way ANOVA with Tukey post-hoc test.

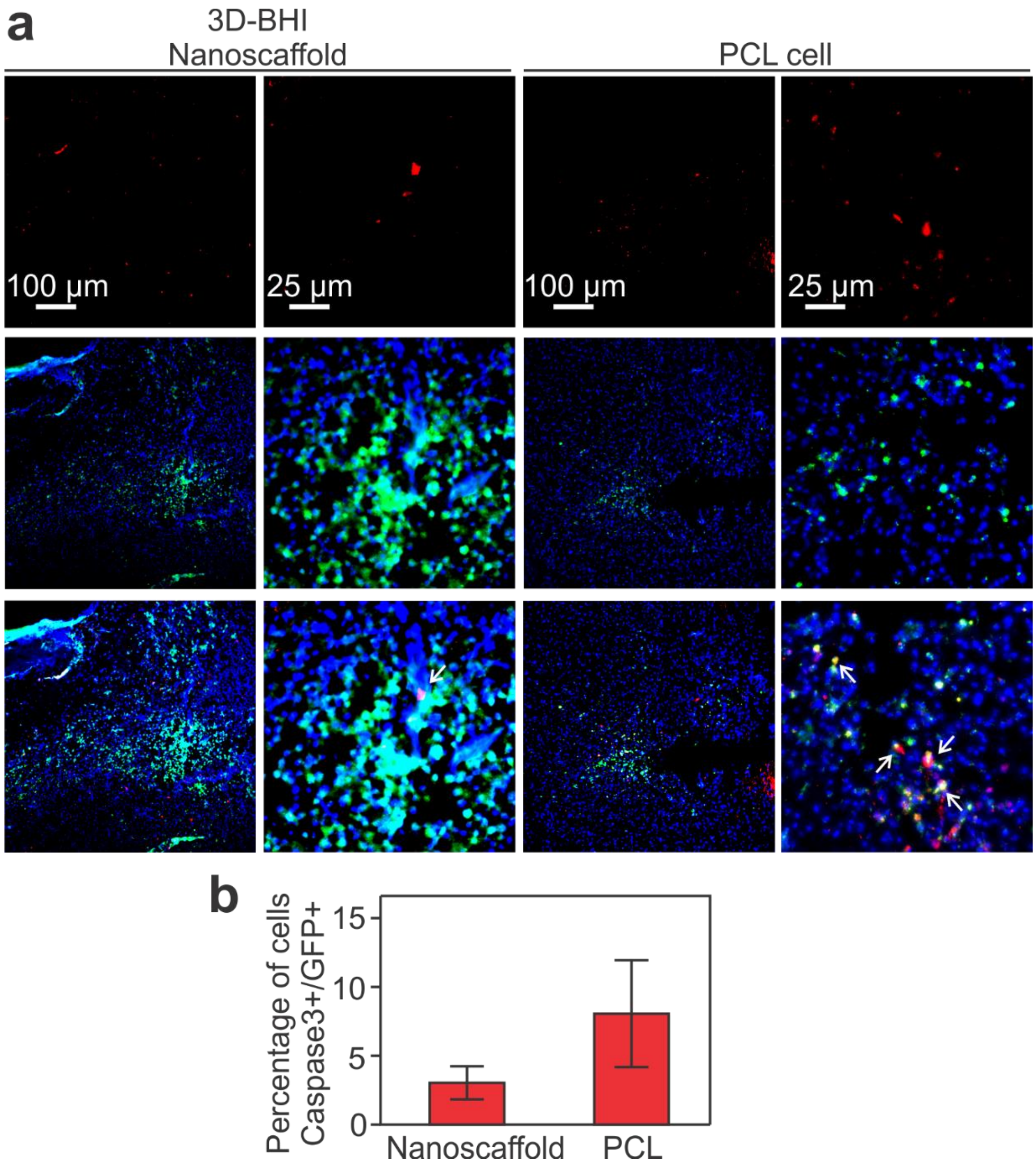

Supplementary Figure 23. Enhanced cell survival of transplanted GFP-iPSC-NSCs on 3D-BHI nanoscaffolds compared to cells transplanted by PCL scaffolds. Tissue analysis was performed 7-day post injury. All tissue sections were from the center of spinal cord injury and nearby the transplantation sites. **a**, Immunohistological staining images of tissue sections from nanoscaffold group and PCL-cell group. Blue indicates cell nucleus (DAPI), green indicates GFP labeled iPSC-NSCs, and red stains cleaved Caspase 3. From the images, higher GFP+ cells and lower percentages of dead cells (Caspase 3 positive cells can be observed in our nanoscaffold group. **b**, Quantifications of Caspase3+/GFP+ cell percentages existent in GFP+ cells. Data represents mean$\pm$s.d., n=3, no significant statistical difference can be detected between the groups (P>0.05) by unpaired student t-test.

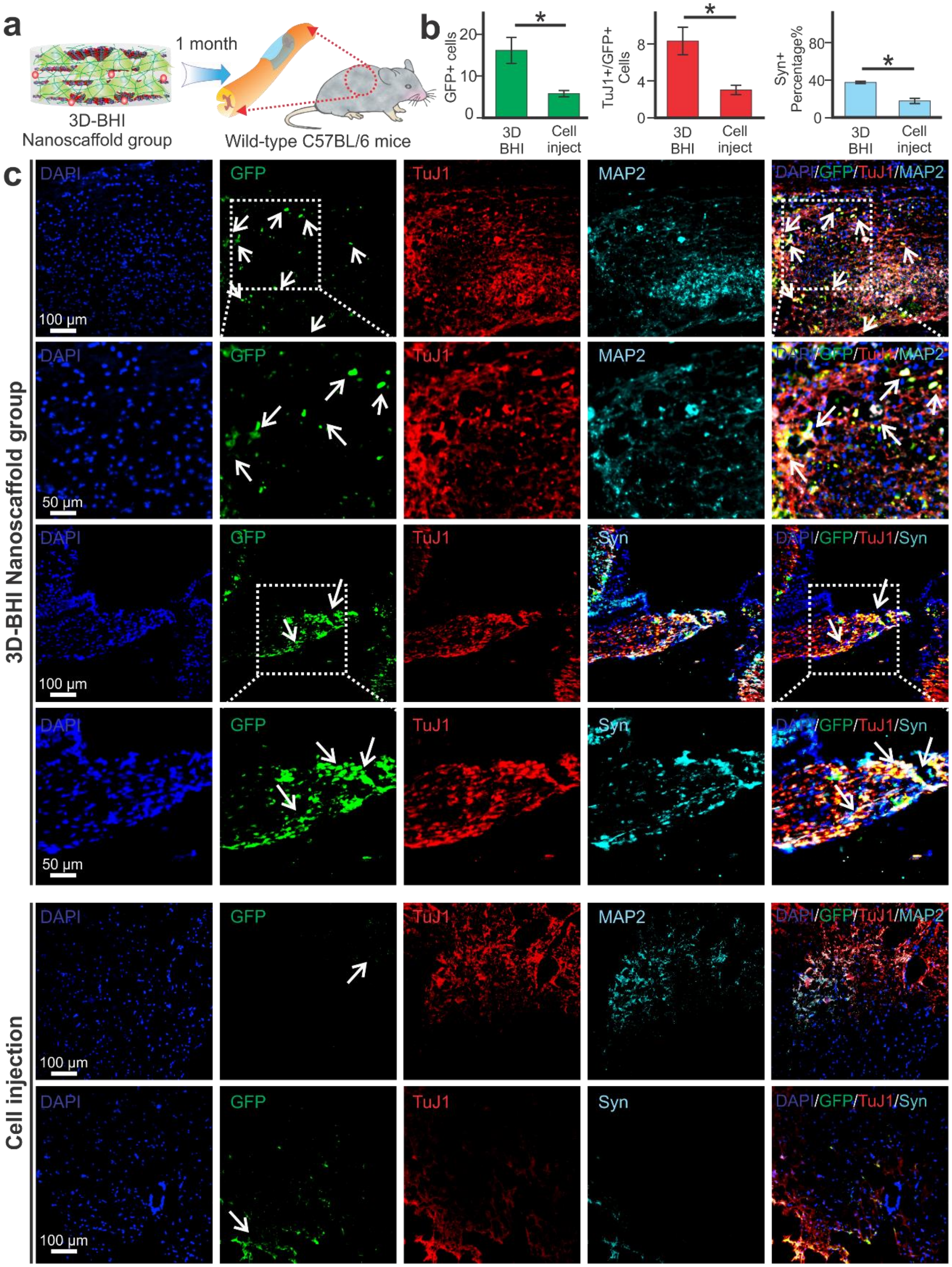

Supplementary Figure 24. Enhanced transplantation and neuronal differentiation of iPSC-NSCs by 3D BHI nanoscaffold 1-month post injury. **a**, Schematic diagram illustrating the transplantation of iPSC-NSC-GFP into injured spinal cord for one month. **b**-**c**, Statistic summary (**b**) and corresponding immunostaining images (**c**) from 3D-BHI nanoscaffold group and cell injection group 1-month post-injury. All tissue sections in **c** were from the center of spinal cord injury and nearby the transplantation sites. GFP, TuJ1 and Syn positive cells were counted in individual sections (674 μm by 674 μm). Data in **b** are mean±s.d. n=3, *P<0.05 by one-way ANOVA with Tukey post-hoc test.

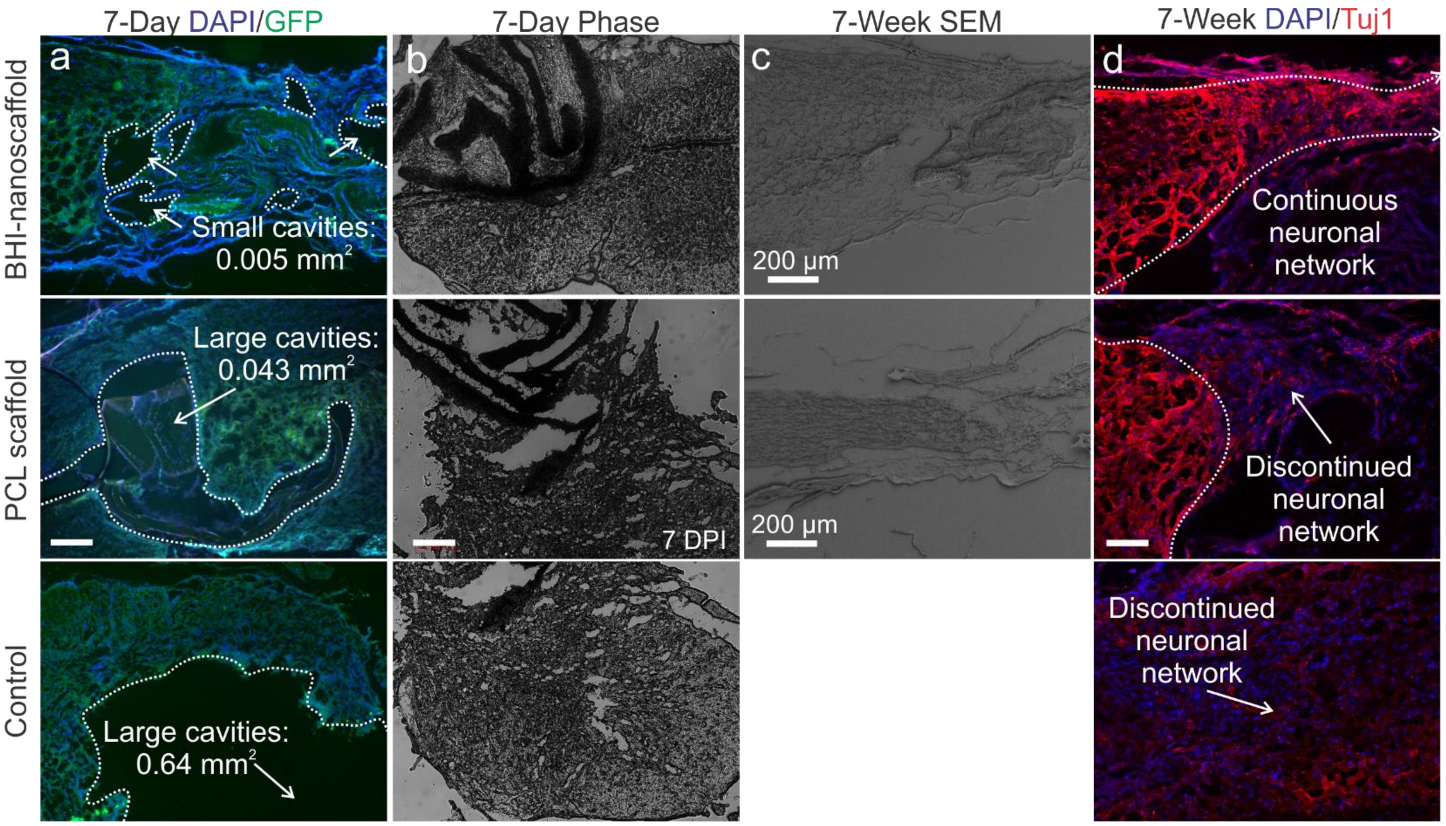

Supplementary Figure 25. Supporting images describing beneficial effects on injured spinal cord from the 3D-BHI nanoscaffolds treated condition compared to the control conditions. a-d, Fluorescence imaging (in row **a**), phase imaging (in row **b**), electron microscopic imaging (in row **c**) and neuronal marker staining in the SCI sites 7 WPI, 7 DPI, 7 WPI and 7WPI, respectively. Different imaging techniques at different time points consistently reveal significantly higher cavities formation and less continuous neuronal networks in the PCL scaffold treated mice or the control groups compared to our nanoscaffold treated groups. Scale bars: **a, b, d**: 50 μm.

SUPPLEMENTARY TABLES

Molecules	Functional group	Binding Energy (kcal/mol)	Intra-molecular Distance (Å)
CH_3Cl	-Cl	-3.55	3.16
H_2O	—	-4.0	3.45
CH_3F	-F	-5.10	2.96
CH_3OH	-OH	-6.43	2.54
CH_3COOH	-COOH	−7. 26	3. 01
CH_3NH_2	$-NH_2$	-10.23	2.98
$Ph\text{-}CH_3$	-Ph	−11. 66	3. 08
DAPT	Drug	-18.43	6.3

Supplementary Table 1. Summary of binding energies (BE) between molecules with assigned functional groups and the 2D MnO_2 nanosheet as computed using DFT calculations. Red fonts indicate the strongest absorption on MnO_2 nanosheets.

Targets (species)	Forward Primer (5' to 3')	Reverse Primer (5' to 3')
GAPDH (Human)	CCGCATCTTCTTTTGCGTCG	GCCCAATACGACCAAATCCGT
TuJ1 (Human)	GGTGTCCGAGTACCAGCAGT	TTCGTACATCTCGCCCTCTT
GFAP (Human)	AGGAAGATTGAGTCGCTGGA	AACCTCCTCCTCGTGGATCT
GAP43 (Human)	AACCTGAGGCTGACCAAGAA	GGGACTTCAGAGTGGAGCTG
FAK (Human)	TGGTGCAATGGAGCGAGTATT	CAGTGAACCTCCTCTGACCG

Supplementary Table 2. Summary of the primers used for quantitative PCR. All primers were obtained from the PrimerBank database.
